# Natural feed additives and bioactive supplements versus chemical additives as a safe and practical approach to combat foodborne mycotoxicoses

**DOI:** 10.3389/fnut.2024.1335779

**Published:** 2024-02-19

**Authors:** Stoycho D. Stoev

**Affiliations:** Department of General and Clinical Pathology, Faculty of Veterinary Medicine, Trakia University, Stara Zagora, Bulgaria

**Keywords:** food safety, mycotoxins, foodborne ailments, preventive measures, risk management, feed additives, biological supplements, herbal additives

## Abstract

This review highlights the possible hazard of mycotoxins occurrence in foods and feeds in regards to foodborne diseases. The possible management of the risk of contamination of foods and feeds with mycotoxins by using natural feed additives, protecting against deleterious effects of mycotoxins or inhibiting the growth of fungi and mycotoxin production, is deeply investigated in the available literature and some effective measures for safe utilization of mycotoxin contaminated feed/food are proposed. The biological methods of decontamination, degradation or biotransformation of mycotoxins are deeply analyzed and discussed. Some natural antagonists against target fungi are also reviewed and a comparison is made with conventional fungicides for ensuring a safe prevention of mycotoxin contamination. The most common and useful chemical methods of mycotoxins decontamination of agricultural commodities or raw materials are also investigated, e.g., chemical additives inactivating or destroying and/or adsorbing mycotoxins as well as chemical additives inhibiting the growth of fungi and mycotoxin production. The practical use and safety of various kind of feed/food additives or herbal/biological supplements as possible approach for ameliorating the adverse effects of some dangerous mycotoxins is deeply investigated and some suggestions are given. Various possibilities for decreasing mycotoxins toxicity, e.g., by clarifying the mechanisms of their toxicity and using some target antidotes and vitamins as supplements to the diet, are also studied in the literature and appropriate discussions or suggestions are made in this regard. Some studies on animal diets such as low carbohydrate intake, increased protein content, calorie restriction or the importance of dietary fats are also investigated in the available literature for possible amelioration of the ailments associated with mycotoxins exposure. It could be concluded that natural feed additives and bioactive supplements would be more safe and practical approach to combat foodborne mycotoxicoses as compared to chemical additives.

## 1 Introduction

Mycotoxins are secondary toxic fungal metabolites, which are well known contaminants of feed and various food commodities and can pose a serious hazard for animals or humans. Contaminated foods and feeds with some mycotoxins can provoke many health ailments in animals/humans, especially in developing countries with lower standards of food quality (1). The invasion of cereals by fungi mainly happens in the field conditions or during the storage. In most cases the production of mycotoxins by fungi is unavoidable due to some environmental conditions such as excessive raining at harvest time or bad storage conditions of grain or feed/food. The mycotoxin contamination of food/feed is often reported to be at a high level. A single fungus or several fungi can produce several mycotoxins leading to multiple mycotoxin contamination in a single food commodity, which pose a serious hazard for health of animals/humans (2, 3). Nowadays, above 400 types of natural mycotoxins are known, but only 10–12 are found to present a serious health hazard for humans or animals, e.g., ochratoxin A (OTA), aflatoxins (AFs) as aflatoxin B1 (AFB1) and aflatoxin M1 (AFM1) are the most dangerous ones, fumonisins (FUMs) as fumonisin B1 (FB1) is the most dangerous one, deoxynivalenol (DON), nivalenol (NIV), zearalenone (ZEA), T-2 and HT-2 toxins, patulin (PAT) and ergot alkaloids, because all these mycotoxins often contaminate human food or animal feed in high levels provoking some human/animal ailments (1, 4, 5). Some mycotoxins are also reported to contaminate animal/chicks products, e.g., eggs, milk and meat, when such mycotoxins are ingested by animals/chicks via contaminated feed (6).

**Figure 1:**
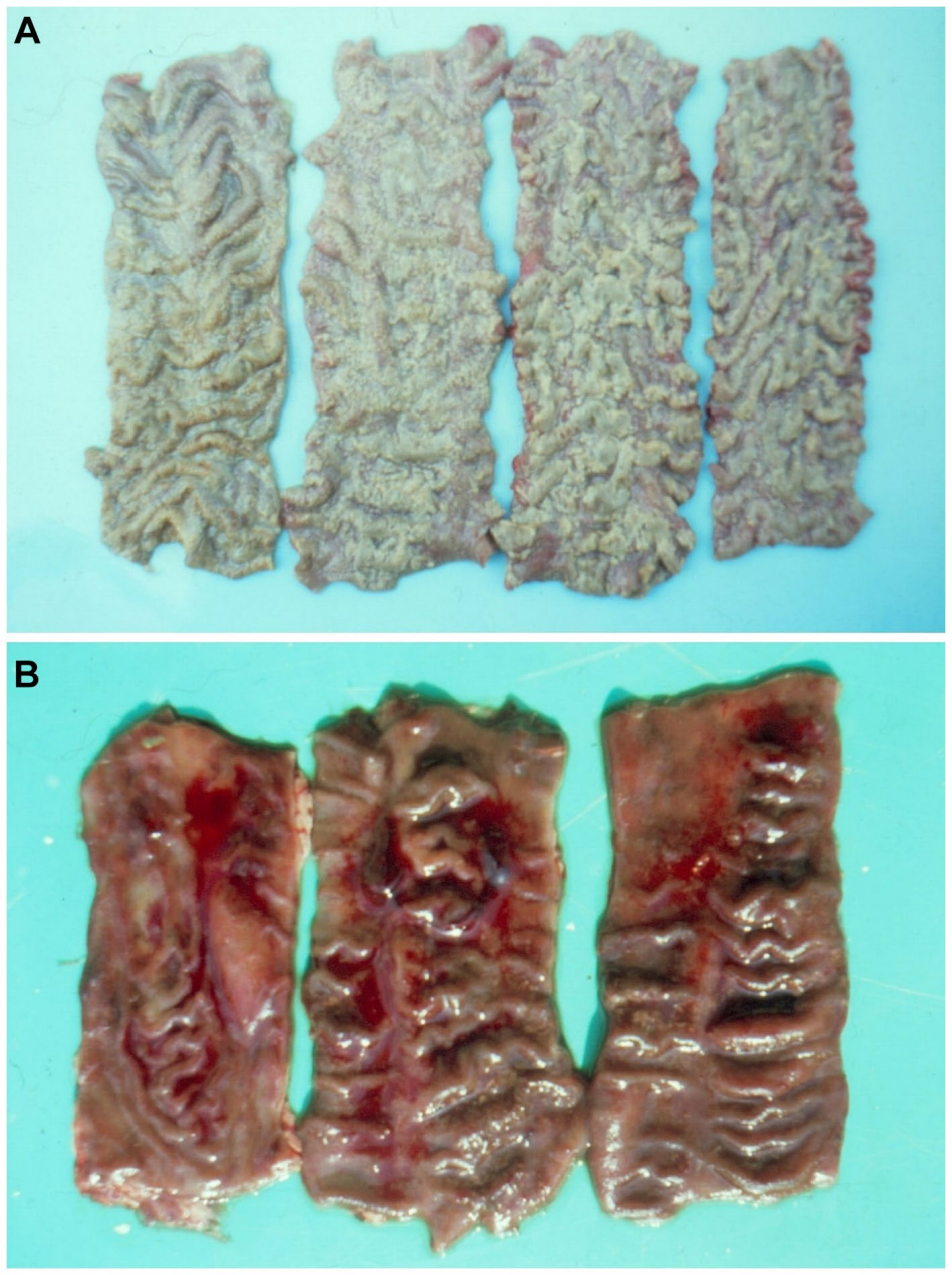
**(A)** Diphtheroid accretions on colon mucosa of a pig given 3 ppm ochratoxin A for 17-days and sick by secondary salmonellosis. **(B)** Haemorrhagic dysentry in pig 47 days after commencing a diet containing 1 ppm ochratoxin A (7).

**Figure 2:**
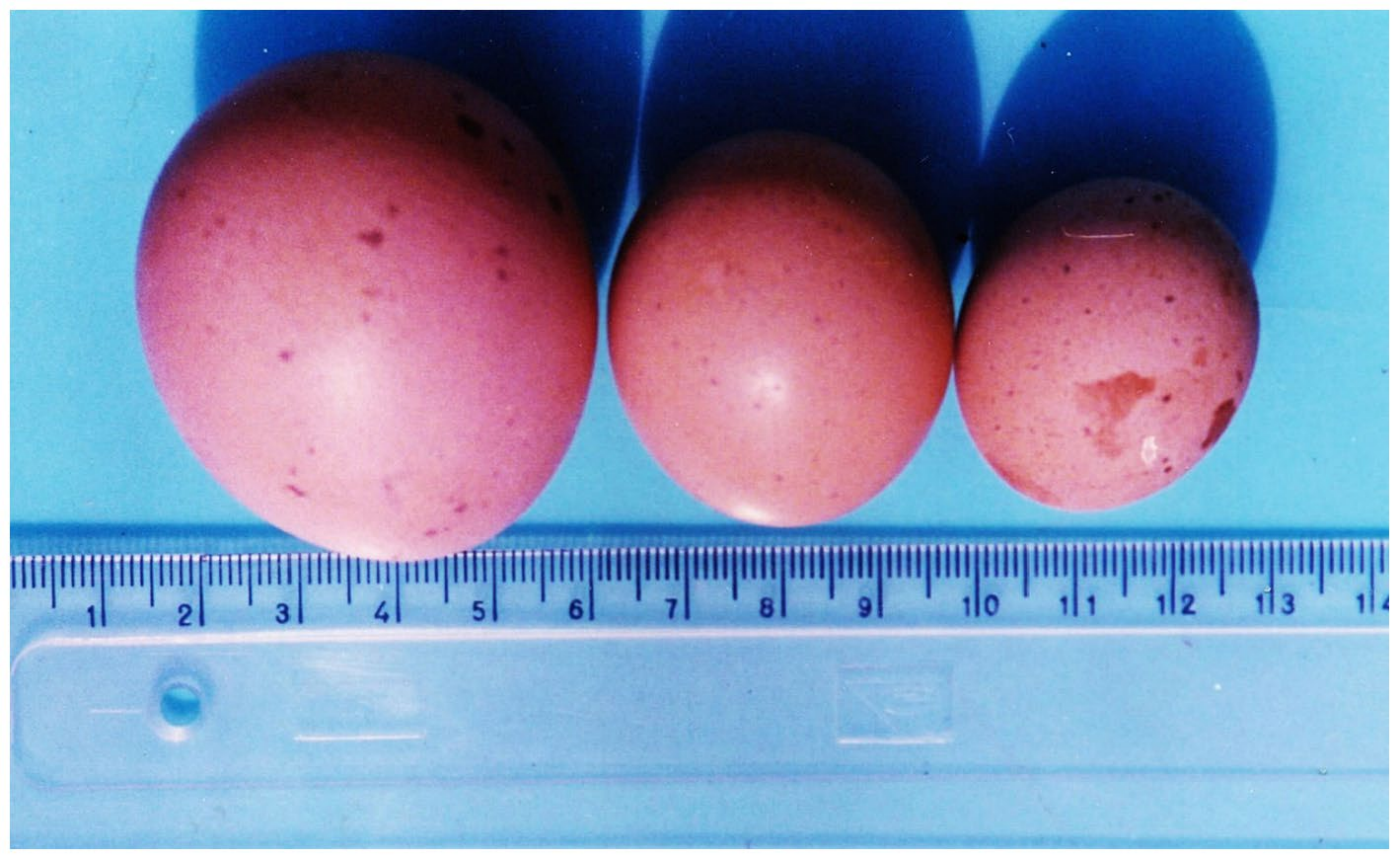
Eggs several times smaller than normal size, weighing 15.8 g and 25.8 g, respectively (from right to left) and varying in size spots and defects on the shell from laying hens exposed to 5 ppm OTA. Left—a normal-sized egg from the control group of laying hens (8).

**Figure 3:**
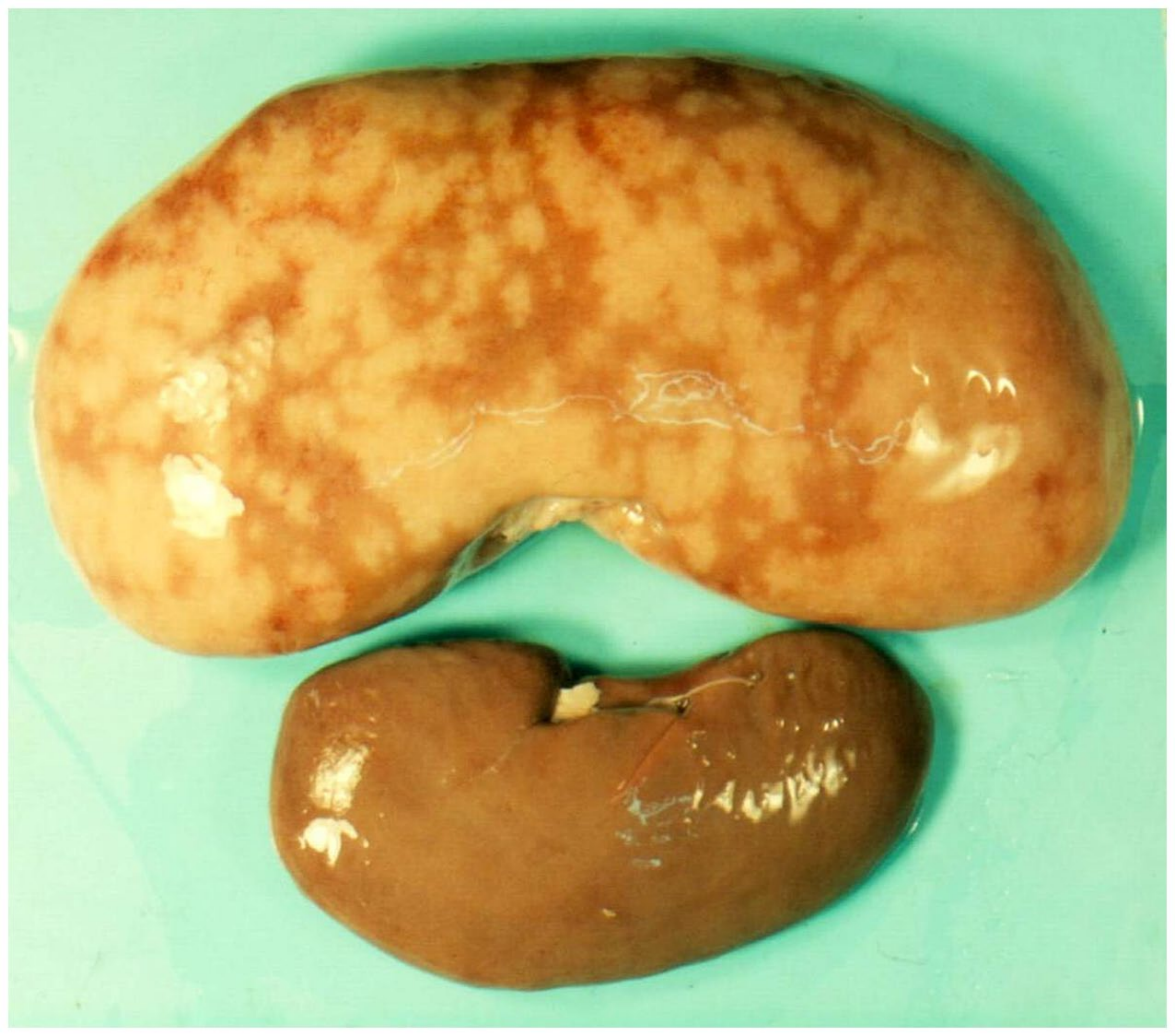
Macroscopic appearance of kidney with spontaneous mycotoxic nephropathy in Bulgaria. Enlarged and marbled appearance of kidney in pig of 6–8 month age (above) and normal kidney in pig of the same age (below) (10).

**Figure 4:**
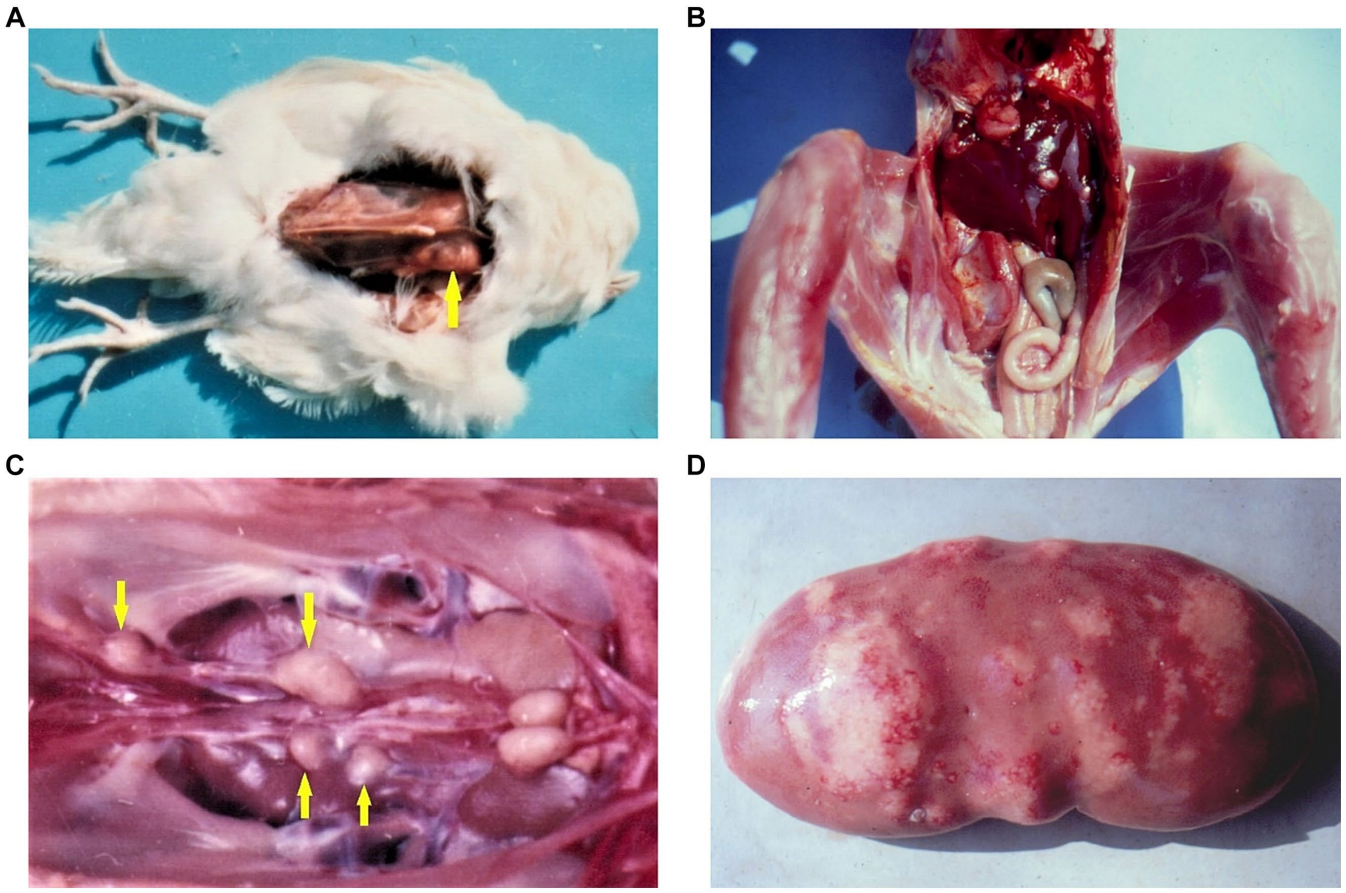
**(A)** Rabdomyoma in the breast muscle (yellow arrow) of female chick exposed to 5 ppm OTA and 25 ppm PHE via the feed, which was slaughtered at the end of the 24th month of the experiment. Large neoplasia in the region of breast muscle, which protruded significantly above the surface. **(B)** Adenocarcinoma in the liver of male chick exposed to 5 ppm OTA via the feed, which died at the end of the 10th month of the experiment. Large grey-white neo-plastic foci in the diaphragmatic surface of the liver, which protruded significantly above the surface. **(C)** Carcinoma in the region of ureters (yellow arrows) of male chick exposed to 5 ppm OTA via the feed, which died at the end of the 20th month of the experiment. Large grey-white neoplastic foci are seen along the ureters and protruded significantly above its surface. **(D)** Neoplastic tissue proliferation (fibroma and fibroadenoma) in kidney with spontaneous mycotoxic porcine nephropathy (10, 13).

**Figure 5:**
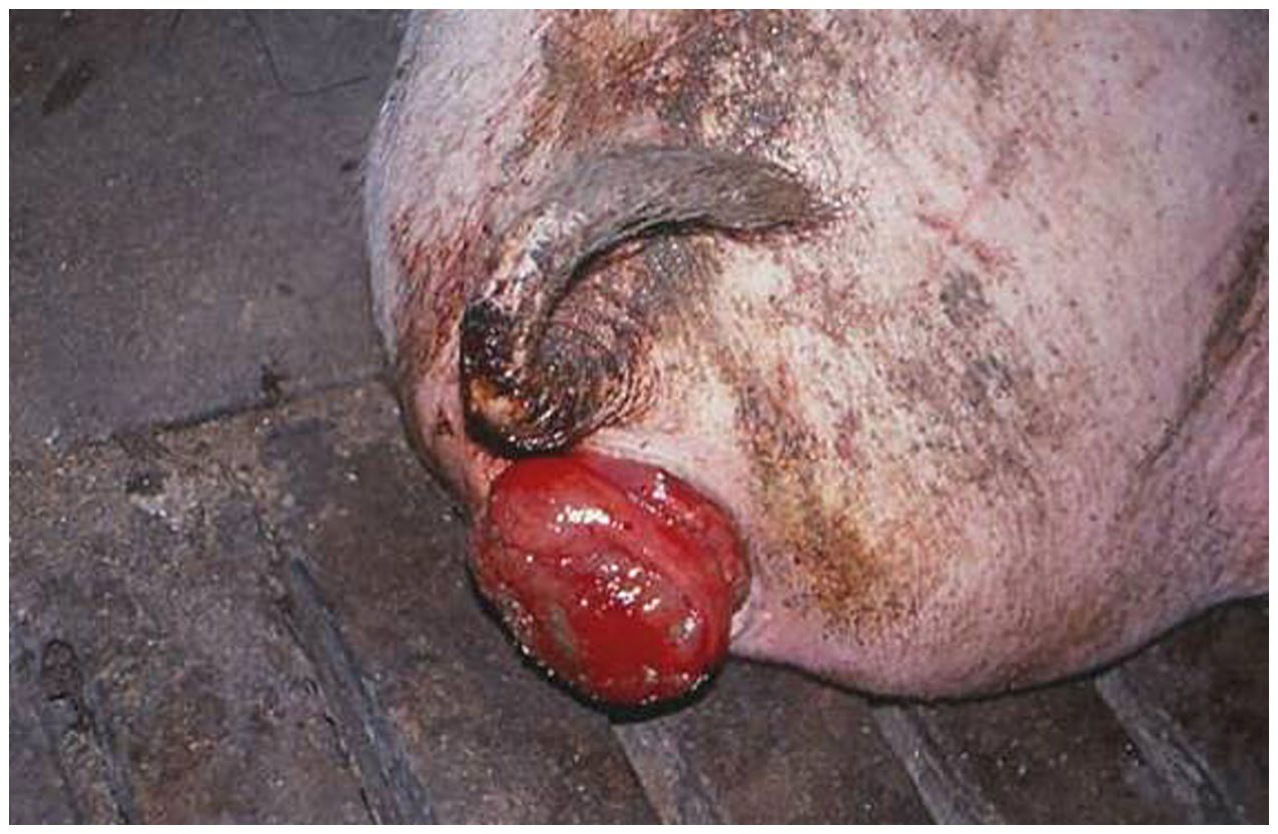
A rectal prolapse in spontaneous case of fusariotoxicosis in pig from Bulgaria due to oestrogenic effect of mycotoxin zearalenone (ZEA) (2).

**Figure 6:**
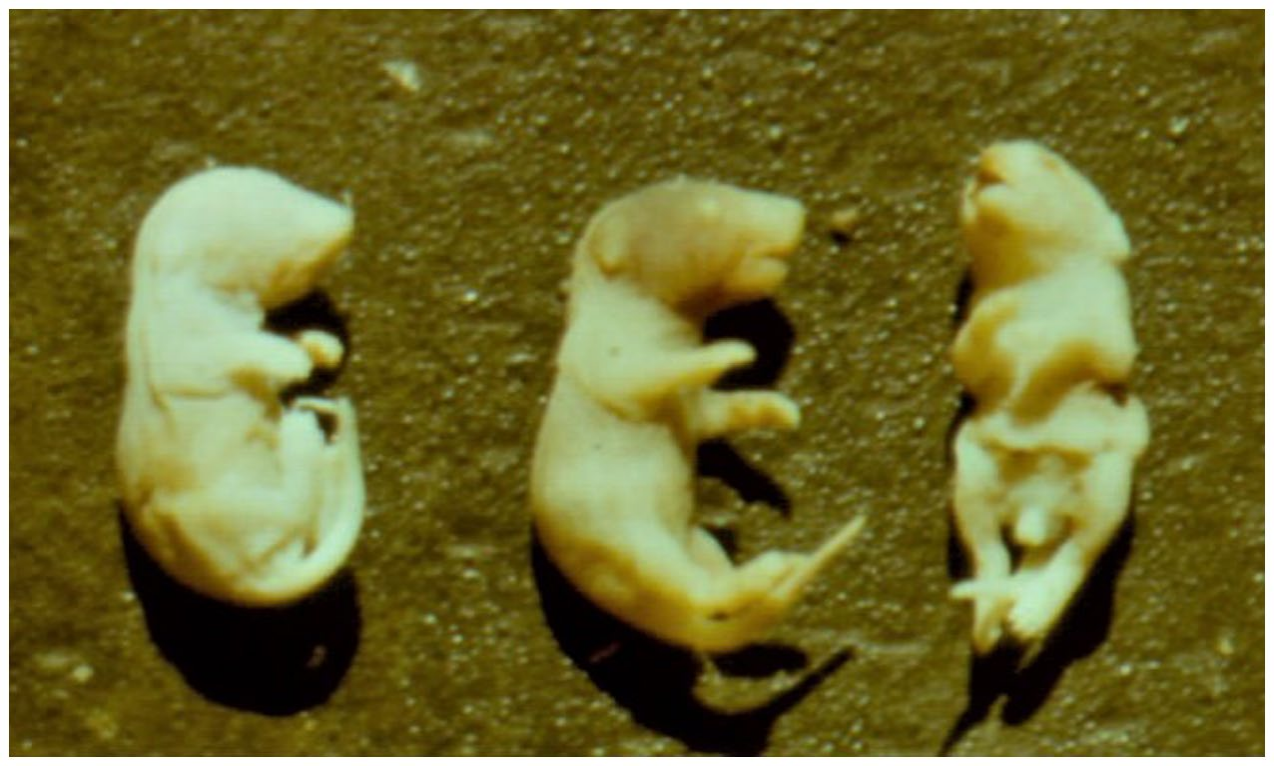
A potent teratogenic effect in newborn mice whose mothers were exposed to 20 ppm OTA (corresponding to about 2.8 mg / kg b.w. per day) and 6 ppm OTB in the feed given from day 7 up to day 12 of the pregnancy—astomia, anophthalmia (on left), normal fetus (in the centre) and spina bifida, e.g., facial cleft and maxillary hypoplasia (on right) (16).

The common health problems, which appear when animals are exposed to mycotoxins via the feeds are: poor feed conversion, feed refusal, decreased weight gain, foodborne ailments, increased secondary microbial infections due to impairment of immunity (Figure 1) (7), and some problems with reproductive and productive capacities (Figure 2) (3, 8, 9). This happens, because mycotoxins can exert various harmful toxic effects (1) such as neurotoxic (FB1 and DON), hepatotoxic (mainly AFB1 and OTA), nephrotoxic (OTA and FB1) (Figure 3) (11, 12), immunosuppressive (mainly AFB1, T-2 toxin and OTA), carcinogenic (mainly AFB1, FB1 and OTA) (Figure 4) (10, 13–15), oestrogenic (mainly ZEA and slightly DON) (Figure 5) (2), genotoxic or teratogenic effects (mainly AFB1, OTA and T-2) (Figure 6) (15–17).

Low mycotoxin contamination levels, which are in the accordance with European regulations (18–22), have been found in many food/feed samples (1), but such multiple mycotoxin contamination in low levels might still exert harmfull effects on animals and humans due to additive or synergistic interactions between some mycotoxins. Such multiple mycotoxin exposure and mycotoxin interaction is often responsible for many foodborne ailments. In this regard, the significance of low content of some target co-occuring mycotoxins in food commodities or feeds for appearance of some foodborne diseases should be additionally studied (1). The adequate risk assessment and possible health hazard for some animals or humans should be additionally elucidated in order to undertake suitable preventive measures in such cases. Natural feed additives as a safe approach to combat deleterious effects of mycotoxins and foodborne mycotoxicoses would be a useful tool in the same circumstances and could contribute for ensuring safe foods or feeds.

According to FAO, nearly a quarter of the world’s crops are contaminated with mycotoxins each year, which is responsible for subsequent annual losses of nearly 1 billion tons of food production (23). Moreover, a lot of people worldwide, especially in developing countries, are exposed to deleterious effects of mycotoxins via the crops, meat, dairy products, coffee, spices, wine, beer and others. That’s why, the social and economic impact of mycotoxins contributes to various kind of losses, because of diseases or death of animals/humans, health problems, increased costs for veterinary service or medical care, decrease in growth and animal performance, decrease in animal produce and livestock production, increased expenses for control and preventive measures, increased research costs and costs for mycotoxins detoxification, feed/food losses due to scrapping, waste products due to mycotoxin content and many others (4). Having in mind, that mycotoxin contamination is the main cause for the above mentioned loses, e.g., condemnation of grain or food commodities, natural feed additives could serve for safe utilization of mycotoxin contaminated feed/food proposing a helthy solution of such problems (2).

The purpose of the review paper is to elucidate the possible management of the risk of contamination of foods and feeds with mycotoxins by using natural feed additives. It will be deeply investigated in the available literature in order to propose some effective measures for safe utilization of mycotoxin contaminated feed/food. The most common and useful chemical or biological methods of mycotoxins decontamination of agricultural commodities or raw materials will be also investigated. The practical use of feed/food additives or probiotics as possible methods for ameliorating the adverse effects of some dangerous mycotoxins will be reviewed and discussed. The efficacy and safety of natural feed additives or biological supplements will be compared with chemical feed additives in regard to possible use as a practical approach to combat foodborne mycotoxicoses. The most dangerous mycotoxins in agricultural commodities and their possible hazard in regards to foodborne ailments will be also elucidated.

## 2 Chemical additives as a method of mycotoxin decontamination or detoxification

### 2.1 Chemical additives inactivating and/or destroying mycotoxins

A lot of chemical substances are reported to inactivate or destroy mycotoxins, e.g.: some bases (such as ammonia or sodium hydroxide), acids (such as propionic or formic acids), chlorinating agents (such as chlorine dioxide, sodium hypochlorite or gaseous chlorine), reducing agents (such as bisulphite), oxidizing compounds (such as hydrogen peroxide or ozone) and some other chemicals such as formaldehyde (24–26). However, it should be underlined, that most of the chemical methods for decontamination of mycotoxins can decrease the nutrient quality of the treated foods or feeds. Also, the usage of chemicals only for decreasing of mycotoxin content in juices is not desirable. Additionally, the residues of some chemicals or their toxic compounds have unacceptable side effects (25, 26). Therefore, their extensive use for mycotoxin decontamination of animal feeds, except ammoniation, is limited and considered as impractical and even as potentially dangerous for largescale use in practice (27). However, such chemical additives could be very fruitful for ensuring a reduction of mycotoxin content in fruits, but can also lead to deterioration on the nutritional qualities of the fruits (28). In this regard, some additional studies are necessary to fully understand the possible degradation of products, which are treated with target chemicals designed for destroying of mycotoxins, in addition to clarification of the required conditions and feasibility when such additives are applied in industrial scale.

Some chemicals such as 0.25% concentration of formic acid can destroy OTA only whithin 3 h treatment, 1% propionic or sorbic acids whithin 24 h treatment and 0.5% benzoic acid whithin 24 h treatment (29).

The chemicals such as sulfur dioxide, hydrogen peroxide, potassium permanganate, ozone, ammonia, and others are found to be very helpful in degradation of PAT. Some of the same chemicals are also allowed in processing of foods within EU (30, 31). Among them the ozone was found to have the greatest potential for PAT degradation in liquid food, but unfortunately it was reported to be very dangerous to human health (32).

The ozone exposure as a gaseous substance is often used for decreasing the content of PAT and *Alternaria* mycotoxins in some fruits (33). The ozone treatment at such a low level as 0.19 mg/L has been found to be enough to eliminate above 98% of PAT (31). Some other studies also report such a potent degradation of PAT by ozone treatment (34–36).

The hydrogen peroxide, which is another potent oxidizing substance, can oxidize AFs into less toxic compounds and is often used for detoxification of peanuts contaminated with AFs, whereas monomethylamine and calcium hydroxide are often used for detoxification of corn and oil-seeds contaminated with FUMs (37).

Similarly, the fruits spraying by a defined solution of hydrogen peroxide is used to decrese the contamination levels of PAT and *Alternaria* mycotoxins (38).

Sodium bisulphite is also a chemical substance, which is helpful for detoxifying of dried figs and corn containing the high levels of AFs or DON (2, 26). Such treatment includes soaking of the respective fruits in a defined solution of sodium bisulfite for target periods of time.

It is also found, that sodium chloride (salt) when used at the time of the cooking of unshelled peanuts under pressure can reduce contamination levels of AFs (2).

The treatment (washing) with sodium hypochlorite solution for different periods of time is another chemical method which is helpful in reducing the content of *Alternaria* mycotoxins and PAT in fruits (39).

In principle, ammoniation and ozonation include chemicals, which could be used in the practice to decontaminate peanuts and feeds containing high levels of AFs or FUMs, but such chemicals are forbidden in European Community (EC) for treatment of human food commodities (2, 26). When OTA or FUMs contaminated grain is treated with ammonia, the content of FUMs and OTA was found to decrease strongly and the mold growth was found to be inhibited (40). Unfortunately, further studies found that the ammoniation process can provoke some unwanted decrease in the nutritional value of the feeds or food commodities, including a reduction of sulphur and lysine containing amino acids (2). Also, a subsequent aeration after the treatment with amonia is required for feedstuffs in order to be accepted by animals.

In another experimental study, it was found that the treatment of barley or grain by 5% NH_3_ or 0.5% NaOH at high temperature can destroy the bigest part of OTA, but usually the same are rarely used, because are not practical (39, 41).

### 2.2 Mycotoxin-adsorbing chemical additives

A good strategy for reducing mycotoxin exposure is the usage of mycotoxin-adsorbing feed additives, which can decrease mycotoxin adsorption and its bioavailability in the respective animals or poults. The adsorbents or binders such as activated carbon and chitosan resins present other useful ways to neutralize mycotoxins in feed/food. For example, the adsorbents such as activated carbons or modified chitosan resins are reported to be helpful in removing PAT from fruit juices (42).

The biggest part of such detoxifying materials are usualy based on mineral clays or other compounds that can adsorb mycotoxins from contaminated feed and to reduce their absorption in gastrointestinal system, and in such a way to facilitate mycotoxin excretion (2). The main substances, which are very useful in mycotoxins binding and subsequent prevention of mycotoxins absorption, usually are with high molecular weight. The formation of adsorbent–mycotoxin complexes increase their fecal excretion and detoxification (43). Such mycotoxin-adsorbing additives could be some silica-based inorganic substances or carbonbased organic polymers. In this regard, bentonite clays such as Hydrated sodium calcium aluminosilicate (HSCAS) or zeolitic minerals present are a large group of aluminosilicates with good possibilities for mycotoxins binding in gastrointestinal tract and subsequently decreasing mycotoxins bioavailability. Such clays are very effective in AFs-contaminated feeds and could decrease AFs transmission from feeds to milk of lactating animals (25, 44, 45). However, such clays may decrease the nutritive quality of agricultural commodities by binding various nutrients together with mycotoxins. It was also found that absorption of AFs in gastrointestinal system could be effectively decreased by bentonite (46). However, the same adsorbents, such as kaolin or sepiolite, similarly to the other clays, are rarely effective in regards to the other mycotoxins such as T-2 toxins, OTA, DON and FUMs (2, 47–51).

The previous studies support the above mentioned statements. For example, OTA absorption in gastrointestinal system is not influenced by HSCAS addition (in 1%) or bentonite addition (in 1% or 10%) to animal diet and no changes are seen in OTA levels in the blood, tissues or bile of treated pigs (52). Also, T-2 toxin could be adsorbed by bentonite, only when bentonite is added at 10 times higher (100 g/kg) level to diet than this one used for elimination of AFs (53). However, 1% activated charcoal added to animal feeds was found to decrease slightly OTA levels in blood of pigs, whereas 10% charcoal was found to decrease significantly OTA levels in blood and various tissues (52, 54). Unfortunately, the addition of charcoal to the animal diet is impractical method of decreasing OTA absorption, because of its high price and the possibility of subsequent decrease in vitamins and minerals absorption in treated animals (52).

Cholestyramine, a commercial anion exchange resin, is another absorbent, which is reported to be effective in various cases of OTA, FUMs or ZEA contaminations in feeds. It was seen to reduce significantly (about 50%) the contamination levels of OTA in blood, when supplemented to OTA containing rat diet (54).

In this regard, the modified chitosan resins or magnetic chitosan are found to be effective in removal of PAT without side changes on the quality of juice (55–59). In comparison to the other methods of removal, the removing mechanism here is very clear and no risk is found of possible toxicity if the correct adsorbent is selected and properly recovered from the juice (60).

Having in mind that clay binders are rarely effective against most of mycotoxins, excluding AFs and PAT, natural organic binders are also investigated for the same purpose. Such organic binders are found to be effective against several mycotoxins, which is useful in the cases of multi-mycotoxin contamination. It is important to underline, that such natural organic binders are highly biodegradable, which prevents environmental contamination (48).

Another possible way for mycotoxin removing from the feed/food is the extraction by solvents such as ethanol, isoprapanol or methoxymethane, which is found to be effective for decontamination of feeds with high levels of AFs, but the high costs and the remaining residues from the solvents put up a barrier, which prevents such extraction methods to be widely used for commercial exploitation (25, 61).

### 2.3 Chemical additives inhibiting the growth and mycotoxin production by fungi

Some other chemicals or antifungal agents such as anthocyanin, polyphenols, and some biologically active substances could also inhibit the fungal growth and mycotoxin production of *A. flavus,* preventing subsequent mycotoxin contamination of feedstuffs (62–64). Such substances would be a more practical way for decreasing mycotoxin contamination of feedstuffs. In this regard, phosphine (PH_3_) was reported to be an effective substance in the suppression of the fungal growth and sterigmatocystin production of *A. versicolor* (65). Methyl paraben and potassium sorbate were also found to be potent antimicrobial agents that would be able to prevent the fungal growth of *Aspergillus* and *Penicillium* species in food/feed with pH values between 5 and 6, e.g., cereals, sorghum or silage and subsequently to prevent OTA production from the same fungal species (66). Potassium sorbate or calcium propionate are also effective against OTA in bread (67).

The combination of cold storage and the treatment of fruit and vegetable with fungicides is commonly used to control postharvest decay for long periods of time. It is reported that annually about 23 thousand tons of fungicides are usually used on a global scale to prevent postharvest damages in vegetables and fruits (68). Some fungicides such as Azoxystrobin are also effective against OTA in wine (69).

A lot of chemical agents are investigated for their protective properties against production of PAT by *P. expansum* or other fungi. The bioactive isothiocyanates packaging at levels of 50 ppb or above is reported to prevent contamination of apples with *P. expansum* (70). The application of exogenous potassium phosphite is found to inhibit significantly the germination of *P. expansum* spores, which are inoculated in pears or apples (71). Beta-aminobutyric acid gamma-aminobutyric acid (72) as well as β-aminobutyric acid (73), are shown to be effective against the growth of this fungus. Hydrogen peroxide and sodium hypochlorite are also successfully tested in the inhibition of spore germination and the growth of *P. expansum* (74, 75). Boric acid (76), sodium propionate and potassium sorbate are also shown to be effective against production of PAT and fungal growth of *P. expansum* (77). The wash treatment with acetic acid in solution levels of 2–5% is reported to inhibit the fungal growth of *P. expansum* in apples (75), whereas the vapor treatment with the same compound in concentration of 6 μL/L is also reported to be helpful in preventing the growth of *Botrytis cinerea* and *Penicillium* species on apples (78).

On the other hand, the addition of some chemicals could also increase the efficacy of some bioactive agents. For example, the addition of some nitrogenous compounds (L-aspartic or L-serine) to *Candida sake* can improve the efficacy of this bioactive agent against contamination of apples with *P. expansum*. Similarly, the bioefficacy of *C. sake* can increase significantly by addition of ammonium molybdate, which can eradicate significantly the blue mold development on pears and apples in cold storage conditions (79). The addition of nitrogenous substanes (L-proline or L-asparagine) can also improve the bioefficacy of *Pseudomonas syringae,* completely reducing the growth of blue mold (80). Some phenolic compounds such as ferulic acid, umbeliferone or quercetin, have been successfully tested on Golden delicious and/or Granny Smith apples (81).

## 3 Plant and herbal additives as a safe approach to combat harmful effects of mycotoxins

### 3.1 Plant and herbal additives inhibiting the fungal growth and mycotoxin production by fungi

It is important to know, that some biologically active substances or plant extracts may act as antifungal agents, e.g., some polyphenols, flavonoids, carotenoids, silymarin, etc., and could suppress the growth of *Aspergillus flavus* preventing possible contamination of feed/food with AFs (63, 64, 82, 83), and could serve as a practical way for preventing mycotoxin contamination.

Some plant extracts were found to be effective in suppressing the growth of PAT- or *Alternaria* producing fungi. In this regard, the essential oils such as cinnamon and clove oil were reported to be useful in decreasing PAT contamination in apples (84). Similarly, the garlic extract usage was seen to be effective in decreasing *Alternaria* mycotoxins in tomatoes. Plants extracts of essential oils and monoterpenoids (85), garlic extract and garlic vapor exposure of apples have been reported to inhibit significantly the growth of *P. expansum* (86).

In this regard, natural antioxidants were found to be very useful for control of fungi at postharvest time and in inhibition PAT production (87) as well as for fungal control and subsequent production of AFs and OTA (88). Some antioxidants such as vanillic acid are effective against OTA synthesis (89).

### 3.2 Plant and herbal additives protecting against deleterious effects of mycotoxins

Another way to reduce deleterious effects of mycotoxins on farm animals and poults is addition of various natural mycotoxin-protecting compounds from plant or herbal origin having protective or antidote effect against some mycotoxins (8, 90–94). In this regard, above 7,000 plant species are currently used in India for medical treatment of some diseases (95) (Table 1).

**Table 1:** Protective effect of some plant/herbal or vitamin additives against deleterious effects of mycotoxins.

Plant/herbal or vitamin additive	Protective effects in animals or poultry	References
5% water extract of Artichoke (*Cynara scolymus L*) given as steam infusion	Improve diuresis and ↑ urinary rout of OTA excretion ↑ hepatobiliary rout of OTA excretion Improve OTA-suppressed eggs production ↑ OTA-suppressed body weight gain ↓ OTA-content in liver and kidneys Protective effects against OTA-induced liver and kidney damages Protective effect against OTA-induced damages in lymphoid organs Vasoconstrictive and permeability decreasing effects against OTA-induced oedematous changes ↓ OTA-increased serum glucose, uric acid, creatinine, urea Protective effect against OTA-suppressed humoral immune response	Stoev et al. (90, 96–98); Stoev (8)
Rosallsat (a plant extract of seminum *Rosae caninae* and bulbus *Allii Sativi*)	Suppress OTA-increased lipid peroxidation Protective effects against OTA-induced liver and kidney damages Protective effect against OTA-induced damages in lymphoid organs ↓ OTA-content in liver and kidneys	Stoev et al. (98)
Roxazyme-G (polyenzyme complement produced by “*Trichoderma*” fungi)	↓ OTA-increased serum glucose, creatinine and urea Protective effects against OTA-induced liver and kidney damages Improve OTA-suppressed eggs production Protective effect against OTA-suppressed humoral immune response ↑ OTA-suppressed body weight gain Protective effect against OTA-induced damages in lymphoid organs	Stoev et al. (97); Stoev (8)
Sesame seed	↓ OTA-increased serum creatinine and urea Protective effects against OTA-induced liver and kidney damages Improve OTA-suppressed eggs production Improve OTA-suppressed protein synthesis Protective effect against OTA-suppressed humoral immune response ↑ OTA-suppressed body weight gain Protective effect against OTA-induced damages in lymphoid organs	Stoev et al. (97); Stoev (8)
Phenylalanine	↓ OTA-increased serum creatinine and urea Protective effects against OTA-induced liver and kidney damages Improve OTA-suppressed eggs production Improve OTA-suppressed protein synthesis Protective effect against OTA-suppressed humoral immune response ↑ OTA-suppressed body weight gain Protective effect against OTA-induced damages in lymphoid organs Protection against OTA-induced carcinogenic effect Protection against OTA-induced teratogenic effect	Stoev et al. (97); Stoev (8, 16, 99, 100)
*Silybum marianum* or Silymarin	Protective effect against OTA-suppressed humoral immune response Protective effects against OTA-induced liver and kidney damages ↓ OTA-increased serum uric acid ↓ OTA-increased enzyme levels of AST and ALT Protective effect against OTA-induced damages in lymphoid organs	Stoev et al. (92, 94)
*Silybum marianum* or Silymarin	↓ AFs-increased enzyme levels of AST, ALT and ALP Protective effects against AFs-induced liver damages ↑ AFs-suppressed body weight gain ↑ in feed conversion ratio in AFs exposed chicks	Tedesco et al. (101); Muhammad et al. (102)
Silymarin	Protective effect against OTA-suppressed humoral immune response	Khatoon et al. (103)
Silymarin	Protection against AFs induced diabetic nephropathy	Soto et al. (104)
*Silybum marianum* or Silymarin	Protective effect against experimental liver damages and the increased levels of AST, ALT, ALP and/or lipid peroxidation in rats/mice Protective effect against oxidative stress	Kaur and Agarwal (105); Pradeep et al. (106, 107); Shaarawy et al. (108); Rasool et al. (109)
Silymarin	Protective effect against experimental kidney damages Protective effect against increase of lipid peroxidation	Karimi et al. (110); Varzi et al. (111)
*Silybum marianum* or Silymarin	Protective effect on humoral and cell mediated immune response Antioxidative effect	Katiyar (112); Kiruthiga et al. (113, 114)
*Withania somnifera*	Protective effect against OTA-suppressed humoral immune response Protective effects against OTA-induced liver damages ↓ OTA-increased enzyme levels of AST and ALT Protective effect against OTA-induced damages in lymphoid organs	Stoev et al. (92)
*Withania somnifera*	Suppress lipid peroxidation Protective effect on liver	Elberry et al. (115)
*Withania somnifera*	Protective effect against brain damages	Schliebs et al. (116)
*Withania somnifera*	Protective effect on humoral and cell mediated immune response Antioxidative effect	Kuttan and Leemol (117)
*Withania somnifera*	↑ of body weight gain Anti-inflammatory effect Anti-neoplastic effect Antioxidative effect Immunomodulatory effect	Mishra et al. (118)
*Centella asiatica*	A slight protection against OTA-suppressed humoral immune response ↓ OTA-increased enzyme levels of AST A slight protection against OTA-induced damages in lymphoid organs	Stoev et al. (92)
*Centella asiatica*	Protective effect on gastrointestinal mucosa, vascular intima and skin Protective effect against oxidative stress	Montecchio et al. (119); Gohil et al. (120)
*Centella asiatica*	Protective effects against liver and kidney damages Immunostimulating effects Anti-bacterial activity	Oyedeji and Afolayan (121)
*Tinospora cordifolia*	Antioxidative effect against OTA provoked oxidative stress Protective effect against the changes in spleen and blood biochemistry in OTA-exposed laboratory animals Protective effect against OTA-induced genotoxic effects	Karamalakova et al. (122, 123)
*Tinospora cordifolia*	Immunostimulating effect Diuretic effect Suppress lipid peroxidation Anti-diabetic, anti-inflammatory, antioxidant, hepato-protective and anti-neoplastic activities	Singh et al. (124); Upadhyay et al. (125, 126); Sharma et al. (295)
*Tinospora cordifolia*	↑ OTA-suppressed body weight gain Protective effect against OTA-induced liver and kidney damages ↓ OTA-increased levels of serume glucose and uric acid Protective effect against OTA-suppressed humoral immune response	Stoev et al. (93)
*Tinospora cordifolia*	Improve humoral and cell mediated immune response	Rege et al. (127); Nagarkatti et al. (128); Sudhakaran et al. (129); Ranjith et al. (130); Upadhyaya et al. (125)
*Tinospora cordifolia*	Hepatoprotective effect Protective effect on gastrointestinal mucosa	Bishayi et al. (131); Panchabhai et al. (132); Sharma and Pandey (133); Kavitha et al. (134); Nagarkar et al. (135)
*Tinospora cordifolia*	Antioxidative effect	Premanath and Lakshmidevi (136)
*Tinospora cordifolia*	Antidiabetic effec expressed by inhibition of alpha glucosidase activity	Rajalakshmi et al. (137); Chougale et al. (138)
*Tinospora cordifolia*	Protective effect against AFs-induced kidney and liver damages Protective effect against AFs-induced oxidative stress	Gupta and Sharma (139)
*Glycyrrhiza glabra*	↑ OTA-suppressed body weight gain Protective effect against OTA-induced liver damages ↓ OTA-increased enzyme levels of AST Protective effect against OTA-suppressed humoral immune response	Stoev et al. (93)
*Glycyrrhiza glabra*	Improve humoral and cell mediated immune response	Mazumdar et al. (140)
*Glycyrrhiza glabra*	Antioxidative effect Suppressive effect on lipid peroxidation	Chin et al. (141); Tohma and Gulcin (142); Latif et al. (143)
*Glycyrrhiza glabra*	Protective effect on liver Anti-oxidative property Decrease serum enzyme levels of AST, ALT and ALP	Al-Razzuqii and Al-Hussaini (144); Rasool et al. (109)
*A. leiocarpus, B. refescens*, *M. oleifera, I. asarifolia* and *G. senegalensis*	Antioxidative effect	Muhammad et al. (145)
*Glycyrrhiza glabra*	Antibacterial/antiviral effect Anti-inflammatory effect Anti-hyper glycemic effect	Kaur et al. (146)
*Glycyrrhiza glabra*	Suppress lipid peroxidation Hepatoprotective effects Hypocholesterolaemic effect Hypolipaemic effect	Sitohy et al. (147)
Polyherbal additive “Growell”	Protection against OTA or AFs induced pathologic damages in internal organs and biochemical changes in blood	Kalorey et al. (148); Sakhare et al. (149)
Turmeric powder	Protective effect against AFB1 induced increase in lipid peroxidation Antioxidative effect in broiler chicks ↓ AFB1 content in the liver of broiler chicks	Amminikutty et al. (150)
Ascorbic acid supplementation to the diet	Protection against toxic effect of OTA on the production and weight of the eggs	Haazele (151); Haazele et al. (152)
Ascorbic acid or vitamin B supplementation	↓ PAT levels in apple juice	Fremy et al. (153); Drusch et al. (30); Yun et al. (154); Meizhen and Ping (32)
Citric acid and sodium bicarbonate given as supplements to the apple juice	↓ PAT levels in apple juice	Kim et al. (155)
Oleanolic acid	Nephroprotective effect against OTA-induced nephrotoxicity Amelioration of OTA-induced apoptotic damages and cell viability in human proximal tubule epithelial kidney-2 (HK-2) cells	Zhang et al. (156)
Ursolic acid	Nephroprotective effect against OTA-induced nephrotoxicity Amelioration of OTA-induced apoptotic damages and cell viability in human proximal tubule epithelial kidney-2 (HK-2) cells Antioxidative effect against OTA provoked oxidative stress	Li et al. (157); Zhang et al. (158)
Vitamin E	Protection against OTA induced immunosuppression	Khatoon et al. (103)

Such herbal feed additives or plant extracts were reported to protect against the suppressing effects of mycotoxins such as OTA on the gain of body weight in stock poults (96–98) as well as on the production of eggs by hens (8). In this regard, such a protection against OTA toxicity and subsequent improvement of OTA elimination from body, was reported for 5% water-extract (prepared via steam infusion) of dried leaves of Artichoke (*Cynara scolymus L*) given via the drinking water or feed of poults at levels 5 mL/kg.b.w. (90, 96–98). The cynarine content in such Artichoke extract can increase the biliary secretion, and improve the hepatobiliary rout of OTA excretion (98). Similarly, the urinary route of OTA excretion is also increased in Artichoke-treated poults due to the improved diuresis and cardiac activity (96, 98). The OTA-content in kidneys and liver in the same studies was found to be lower in poults given Artichoke-extract together with OTA in comparison to poults only given the same contamination level of OTA in feed (97). In addition, permeability decreasing and vasoconstrictive effects of Artichoke extract (96, 98) could decrease OTA-induced oedematous changes in internal organs. The known hepatoprotective effects of cynarin and flavonoids in such water extract of Artichoke could also improve the liver damages in poults due to OTA-exposure (96, 98). The improved diuresis by artichoke-extract could be responsible for amelioration of OTA-provoked increase in serum glucose in the same experimental study (96).

A protective effect was also reported against OTA toxicity in poults for Rosallsat (a plant extract of seminum *Rosae caninae* and bulbus *Allii Sativi*) given as a supplement to the feed of poults in 0.6 mL/kg b.w. daily (98). It was suggested that the bioactive substance allicin and the high levels of some vitamins (e.g., E, A and F) in such a plant extract are responsible for decreasing the toxic effects of OTA (98). The Rosallsat was previously found to suppress lipid peroxidation (98), but the enhanced lipid peroxidation is known to be an important mechanism of OTA toxicity (159). Such increased levels of lipid peroxidation in OTA-treated poults may lead to damages in the cell membrane, which could be responsible for the increase of cellular calcium and subsequent changes in metabolic activity and necrosis of the cells (160).

Another natural bioactive feed additive Roxazyme-G (polyenzyme complement produced by the “*Trichoderma*” fungi) supplemented to diet at level 0.2 g/kg feed was also reported to protect against deleterious effects of OTA in poults (97). The found protection of the same bioactive additive against OTA-provoked increase in serum glucose was explained by the improved energy metabolism (97).

The protection of another natural feed additive “sesame seed “against OTA-induced suppression of humoral immune response and deleterious changes in differential WBC count was explained by the improvement of OTA-induced suppression of protein synthesis, and subsequent improving of the division of the immune cells (97). It was also explained that sesame seed contains a high quantity of phenylalanine, which is known as a structural analog to OTA and, therefore, found to be a good antidote against this mycotoxin (97).

The authors made a conclusion that some of the above mentioned feed additives could serve for a safe utilization of OTA contaminated feedstuffs for poults avoiding possible condemnation of such feedstuffs (90, 96–98).

Protective properties of some herbs, e.g., *Withania somnifera, Silybum marianum* or *Centella asiatica,* given as feed additives at levels of 4,000 ppm, 1,100 ppm, and 4,600 ppm respectively, were reported against the immunosuppressive and toxic effects of OTA in broiler poults immunized against Newcastle disease and exposed to 5 ppm OTA via the diet (92). In the same experimental study, the strongest protection of *S. marianum* and *W. somnifera* was found against immunosuppression and harmful effects of OTA on liver and kidneys. However, the hepatoprotective effect against OTA-induced damages was found to be stronger in poults protected by or *S. marianum* or *W. somnifera,* whereas the protection against kidney damages was better expressed in the poults protected by *S. marianum* as supported by the relative organs’ weight, macroscopic, biochemical and pathomorphological findings (92). The authors claim that the same herbs could be used as a practical means to combat ochratoxicosis and for safely utilizing of OTA-contaminated feedstuffs (92). In the same study it was found, that the investigated herbs possess different mechanisms of protection against OTA toxicity: *W. somnifera* and *S. marianum* were found to be very good immunoprotectors; *S. marianum* was seen to be a good protector against the toxic damages provoked by OTA on liver and kidneys, whereas *W. somnifera* was seen to ameliorate mainly the hepatotoxic damages provoked by OTA (92).

In another study a protection of herbal feed additive Silymarin (standardized seed extract of *S. marianum,* known as Milk thistle) against OTA-induced disturbances in blood biochemistry or kidneys and liver damages were found as measured by the serum concentrations of glucose, uric acid and the activity of enzymes ALT and AST (94).

In another experiment, a strong hepatoprotective effect of *S. marianum* was also found against AFs-provoked liver damages as measured by the decrease of serum enzyme activities of alanine-aminotransferase (ALT), aspartate-aminotransferase (AST) or alkaline phosphatase (ALP) in AFs-intoxicated chicks (102). These enzymes are known as sensitive biomarkers for liver damages. Similar hepatoprotective effect against AFs-induced liver damages in poults is also found in another study (101). In the same studies a better gain in body weight was found in poults fed on diet supplemented with *S. marianum* or Silymarin and additionally treated with AFs (101, 102). In the same experiments, it was found that the feed conversion ratio and the gain in body weight were increased when such chicks were supplemented by *S. marianum* and the measured indices were not different when compared to poults supplemented only by AFs-binder (102). Such an increase in the gain of body weight was also seen in rats supplemented with *W. somnifera* (118).

A similar protection of *S. marianum* was also found in rats with experimental damages of liver as measured by the decreased levels of ALT, AST, ALP and lipid peroxidation (108). A protective effect of *S. marianum* on liver was also seen in mice with hepatic damages due to alcoholic treatment as measured by the inhibition of lipid peroxidation and/or tumor necrosis factor (TNF) and/or the enzyme activity of ALT (105), and in diethylnitrosamine-provoked damages in the liver of rats (106, 107). It seems that the chicks and animals supplemented by the both herbs were found to utilize the feed in a better way.

Similar effect was also reported for polyherbal additive “Growell” given as supplement in experimental ochratoxicosis or aflatoxicosis and in combined mycotoxin exposure of poults (148, 149).

A potent protection of *W. somnifera* and *S. marianum* on cell mediated or humoral immune response was also reported in some other studies (112, 117).

In other studies, a significant protection of the herb *Centella asiatica* was reported on the mucosa of gastrointestinal tract and on the integrity of vascular intima (119, 120), which could protect against the harmful effects of some mycotoxins such as DON or OTA on the mucosa of gastrointestinal system and on permeability of vessels (92). The same herb is suggested to be useful against oxidative damages in oxidative stress as well as against possible damages in the integrity of the mucosa of gastrointestinal system (119, 120), which is often destroyed by some mycotoxins (1). A protection of *C. asiatica* was also reported against some damges in the kidneys or liver in addition to its immunostimulating effect (121), but the same protection was not so strong in experimental ochratoxicosis in poults (92).

A protection power of Silymarin was also reported against AFs-provoked diabetic nephropathy (104) as well as against kidney damages and increase in lipid peroxidation in rats provoked by cisplatin (110) and also against kidney damages in dogs provoked by gentamicin (111).

A dose-dependent protection of Silymarin against OTA-provoked immunosuppression is also reported in poults. In the same experimental study, Silymarin and/or Vitamin E alone or together decreased the immunotoxic effects provoked by OTA, but did not show a significant protection in OTA treated poults in contamination feed levels above 2 ppm (103).

The *S. marianum* or Silymarin used in the above mentioned experimental studies were also reported to have pronounced anti-oxidative properties and a significant protection on internal organs, e.g., immunostimulatory, membrane-stabilizing, nephro- or hepato- protective and liver repairing effects (92, 94, 101, 102, 106, 107, 112, 161–166).

The protective mechanism of *S. marianum* or its seed extract Silymarin was supposed to be a consequence of suppressed lipid peroxidation and/or the elevated levels of endogenous antioxidants, which have beneficial effects on the integrity and the function of cell membranes, and therefore, preventing cell damages due to the enzymes’ leakage, which can destroy the cells (106, 113, 114, 164). The antioxidative property of Silymarin or *S. marianum* could be explained by their suppressive effects on the production of free radicals during the metabolism of toxic substances as well as to elevated levels of hepatic glutathione and the enchanced antioxidant defense of liver (167). The protective properties of Silymarin could be explained by the high quantity of flavonoids, among which, silybin is known to possess the strongest biological activities, incl. Hepatoprotective and nephroprotective activities against various toxic agents (165, 166, 168).

The other herbal additive *W. somnifera* was also found to possess similar organoprotective, antioxidative and immunostimulating properties (117) and suppressive effect on lipid peroxidation in various farm- or laboratory animals, e.g., protective effect on the liver (115), on the nervous system (92, 116). This protection is explained by some bioactive compounds, e.g., steroidal lactones, alkaloids or saponins (118).

Obviously, the same herbal additive Silymarin or *S. marianum* and *W. somnifera* could be used as feed supplements, “in addition to” or “instead of” mycotoxin binders, for decreasing the harmful effects of mycotoxin contamination of the feed in the commercial poultry farms (101, 102, 169). Such herbs could ensure a safe and practical approach for protection of pouls against the toxic properties of mycotoxins such as AFs, FUMs or OTA, and simultaneously can ensure of safe utilization of mycotoxin contaminated feedsuffs while avoiding the respective loss of body weight or condemnation of such mycotoxin contaminated feedstuffs (1).

*Tinospora cordifolia* protection against OTA-provoked spleen and blood toxicity was reported in male ICR albino mice (122, 123). A lot of mycotoxins can provoke oxidative stress (OS) (123, 170, 171) and, therefore, could additionally make worse the health of animals or humans. The protective effect of some herbal additives such as *T. cordifolia*-extract is found to be related to its good antioxidant potential against OS and could find application as protectors against mycotoxicoses (122, 123). The protection of *T. cordifolia*-extract against oxidative stress was reported to be atributed to its good radical scavenging properties against ROS (reactive oxygen species) and RNS (reactive nitrogen species) (172), which usally are increased under the exposure of mycotoxins such as OTA (122, 123). The scavenging properties of *T. cordifolia*-extract against reactive oxygen and nitrogen species (ROS/RNS) were explained by the presence of tannins and phenolic substances (173). The *T. cordifolia*–extract was also found to decrease genotoxic potential of OTA, e.g., 8-OHdG (8-hydroxy-2′-deoxyguanosine) genotoxic biomarker, followed by a decreased level of oxidative activity and gradual recovery of ROS-provoked damages in DNA (122, 123). On the other hand, *T. cordifolia*-extract was found to stimulate activation and differentiation of immune effector cells, e.g., T and B cells, and to increase bile secretion (125), to have diuretic effects (126), and to inhibit lipid peroxidation (124). Therefore, such *T. cordifolia*-extract can additionally improve hepatobiliary and urinary excretion of OTA, to suppress the lipid peroxidation enchanced by OTA, and to stimulate humoral and/or cell mediated immune response, which is usually disturbed by OTA.

Protective effects against OTA-provoked suppression on the gain of body weight and accompanying changes in internal organs and blood biochemistry were also seen for the herbs *Glycyrrhiza glabra* and *T. cordifolia* given as feed supplements to the diet of chicks (93). The decrease in body weight and relative organs’ weight, and the decrease of antibody titer in chicks vaccinated against Newcastle disease were less pronounced in OTA-exposed poults supplemented additionally with *Glycyrrhiza glabra* or *T. cordifolia* in comparison to the poults without herbs supplementation (93). A protective effect of both herbs was also seen against liver damages, but the protection was better expressed in the poults additionally given *Glycyrrhiza glabra* via the feeds as confirmed by pathomorphological changes and lower enzyme activity of AST compared to chicks treated only with OTA. A protection of *T. cordifolia* on kidneys and bone marrow was also observed as seen from the lower serum concentrations of uric acid compared to chicks treated only with OTA (93).

The extracts of *T. cordifolia* (130) and *Glycyrrhiza glabra* (140) were reported to stimulate the both humoral and cell mediated immune response and to improve antibody production *in vivo*. According to some authors, *T. cordifolia* was seen to stimulate phagocytic activity without influencing the humoral or cell-mediated immune immune response (127, 174). However, the same herb was also reported to improve activation and differentiation of T and B cells as well as cytokine production (125, 129).

The Liquorice *(Glycyrrhiza glabra)* is another herb, which is often used in Eastern or Western herbal medicine (146), because of its potent antioxidative and hepatoprotective properties (143, 144), and the immunostimulating potential (93, 140). The protective potential of *Glycyrrhiza glabra* is attributed to some bioactive substances, e.g., flavonoids, glabridin, hispaglarbidin B, isoliquiritigenin, saponin glycyrrhizin, licocoumarin and others (141). The Liquorice has been also reported to possess natural anti-inflammatory (incl. Antibacterial and antiviral), hepatoprotective, cardiotonic, antithrombotic, expectorant and even antidiabetic properties (109, 146) and, therefore, could protect against hepatotoxic, pulmotoxic and immunosuppressive action of some mycotoxins such as AFs, FUMs, OTA or DON (93).

*T. cordifolia* was also found to possess similar imunostimulating, diuretic, anti-inflamatory, hepatoprotective, anticarcinogenic and antidiabetic properties as well as possibilities to suppress lipid peroxidation (126, 128, 131, 175), which could explain its protective properties against hepatotoxic, nephrotoxic, cancerogenic or immunosuppressive action of some mycotoxins and to facilitate their excretion via the kidneys. It was widely used for the treatment of chronic diarrhoea or dysentery and urinary diseases (175), which have been often found in mycotoxin exposed animals.

It was reported, that the both herbs *T. cordifolia* and *Glycyrrhiza glabra* have a strong antioxidative potential, in addition to immunostimulating and organoprotective effect (136, 141, 142), and to be suppressors of lipid peroxidation (147). Therefore, the same herbs could be the good protectors against the toxic effects of some mycotoxins such as OTA, e.g., the increased lipid peroxidation and the oxidative stress (123, 176), the liver and kidney damages, and the immunosuppression (7, 93, 96–98).

The intimate mechanism of protective effect of *T. cordifolia* and *Glycyrrhiza glabra* in OTA-treated chicks (93) could be attributed to the observed decrease of lipid peroxidation, which is enhanced in OTA-treated chicks/animals (7, 159) as well as to the elevated levels of endogenous antioxidants ensuring cellular membrane integrity and preventing the increase of some target enzymes in the cellular cytoplasm and subsequent death of the cells. The immunosuppressive properties of mycotoxins can lead subsequently to some carcinogenic effects, because the important function of natural killer cells is the regular destroying of tumor cells (13, 99, 177). The both herbs were reported to have strong anti-bacterial or anti-viral properties and to be immune boosters (92) and, therefore, could prevent some secondary bacterial diseases, which are often induced by the immunosuppressive properties of mycotoxins (7, 96). These herbs were found to be helpful in some kidney or liver ailments (109, 135) and would be able to protect against OTA/AFs-provoked liver or kidney damages in poults or pigs.

The extract of *T. cordifolia* was reported to inhibits alpha glucosidase, which can explain its anti-diabetic effect (137, 138) and decreased levels of serum glucose (173). The same herb was found to decrease the serum glucose in poults treated with OTA and additionally supplemented with *Tinospora cordifolia* (93), which ameliorate the OTA-induced increase of serume glucose.

*T. cordifolia* is also found to have a strong protective activity on liver (135), to decrease the liver damages provoked by carbon tetrachloride (131, 134), and to ameliorate the liver damages induced by bile salts (127) or lead nitrate (133), and to suppress lipid peroxidation (124, 136). Therefore, *T. cordifolia* could protect against the damages in internal organs provoked by the OTA (93).

*T. cordifolia* was also seen to have good protective properties against AFs-provoked liver and kidney damages (139). *T. cordifolia* was seen to possess protective properties on gastrointestinal system (132), which is possibly realized by protecting against the damaging properties of free radicals on gastrointestinal mucosa (123). Therefore, the same herb could ameliorate the deleterious effects of mycotoxins such as OTA (93) or DON on intestinal mucosa.

It seems that *T. cordifolia* and/or *Glycyrrhiza glabra,* in addition to Silymarin or *Silybum marianum* and *W. somnifera,* could be also used along with some mycotoxin binders for minimizing deleterious effects of mycotoxin contaminated feeds and ensuring a better feed utilization and a higher body weight of commercial poults. According to some authors, such herbs could be used in the practice for realizing a safe utilization of OTA-containing feedstuffs (92, 93) as supported by the improved body weight gain and feed utilization of such poults. In such a way, the economic loss from condemnation of mycotoxin contaminated feeds and weight loss of animals/chicks could be avoided with minimal costs to purchase the same herbs (3, 92, 93). Having in mind the known polarity of most therapeutic substances in herbs, the same authors suggested to use the polar solvents for extraction of such compounds (93). However, some additional efforts are necessary for their application in animal/chicken feeds.

Oleanolic acid, which is often found in various medicinal plants, fruit skins and food materials, was reported to have a nephroprotective effect and poteintial to counteract OTA-induced nephrotoxicity, if given as feed additive. The pre-treatment of 2 μM oleanolic acid for 2 h was reported to ameliorate OTA-induced apoptotic damages and to improve cell viability in human proximal tubule epithelial-originated kidney-2 (HK-2) cells (156).

Ursolic acid, which is a water-insoluble pentacyclic triterpene bioactive compound, is also seen in a lot of medicinal plants and food materials such as cuticular waxes of edible fruits. The same bioactive substance was found to have a nephroprotective possibility against nephrotoxic effects of OTA (158). It was found that a 2 h-pre-treatment with 4 μM ursolic acid could significantly ameliorate mitochondrial-mediated apoptosis in human proximal tubule epithelial-originated kidney-2 (HK-2) cells, induced by 24 h-treatment of 5 μM OTA (158). In another similar study, cell viability, reactive oxygen species (ROS) production, and several proteins’ expressions of human embryonic kidney 293 T (HEK293T) cells were investigated in response to the treatment with ursolic acid and OTA in order to clarify the protective mechanism of ursolic acid against OTA-induced renal cytotoxicity. It was found that oxidative stress was involved in both the nephrotoxicity of OTA and the renoprotective effect of ursolic acid. Results indicated that a 2 h-treatment of 1 μM ursolic acid could significantly alleviate the ROS production and cell death induced by a 24 h-treatment of 8 μM OTA in HEK293T cells (157).

Having in mind that oxidative stress plays a major role in AFB1 toxicity, natural products such as turmeric powder are increasingly being used as an alternative to mineral binders to ameliorate AFB1 toxicosis in farm animals or poultry (150). It was found that broilers exposed for 10 days to AFB1 at dietary levels of 0.02 mg/kg feed showed a significant increase in lipid peroxidation in the liver, which was completely reverted by the concomitant administration of turmeric powder given at feed levels of 400 mg/kg. It was experimentally proved that turmeric powder counteracted such negative effects and simultaneously increased the hepatic gene expression of some antioxidant enzymes (e.g., CAT and SOD2) and decreased the liver content of AFB1 to undetectable levels. The authors suggested that turmeric powder could be an effective feeding strategy to ameliorate AFB1 related adverse effects in broilers (150).

A potent antioxidative effect in a dose-dependent manner was also found for methanolic extracts of *A. leiocarpus, B. refescens*, *M. oleifera, I. asarifolia* and *G. senegalensis.* The phytochemical investigation revealed the presence of alkaloids, flavonoids, and tannins in the same plants, but the phenolic and proanthocyanidin contents of methanolic extracts were significantly higher as compared with aqueous extracts. The authors concluded that DPPH-free radical scavenging activity of methanolic extracts of *A. leiocarpus* and *M. oleifera* was similar to vitamin C and was better expressed as compared with *B. refescens, I. asarifolia, G. senegalensis* (145). A similar antioxidative effect and DPPH-free radical scavenging activity was also reported for methanolic extract of *Desmodium ramosissimum,* which could be another rich source of natural antioxidants, justifying its pharmacological use in traditional medicine (178).

In this regard, it should be mentioned, that there are a lot of similar investigations and reports for antioxidative effects of many plants or plant extracts, but unfortunately the same are not investigated as possible protectors against the toxic effects of mycotoxins in the real practice.

It is interesting to mention, that AFs levels can be significantly decreased in some target kinds of corn processings, such as treatment of maize with lime water in the process of tortillas production (37). A synergistic interaction is also reported in the process of AFB1 degradation between citric acid, lemon juice, and heating of AFB1-contaminated pistachios, because the same mycotoxin is easily destroyed by frying with citric acid and lemon juice. However, such a treatment can change the desired physical properties of the product (179).

## 4 Biological supplements given as feed additives to prevent mycotoxin contamination and to promote mycotoxin degradation

Some other feed supplements/additives can alter the mode of action of mycotoxins via participation of enzymes or live micro-organisms which are involved in biotransformation and detoxification of mycotoxins. Such additives usually receive a great attention from the feed industry, because the same can ensure a promising and safe strategy for preventing mycotoxin exposure, often by reducing mycotoxin bioavailability (2). The action of biological supplements used for preventing mycotoxin contamination or detoxification include the usage of microbial antagonists with fungicidal properties and biotransformation or degradation of mycotoxins by bio-transforming agents such as live and dead microbial cells, enzyme, proteins and culture extracts of yeasts, that are less or non-toxic when ingested by animals and can be easily excreted from the organism (180). Biological detoxification methods usually propose a better safety and flavor of treated food/feed, a preservation of nutritional quality, better organoleptic properties of treated food/feed, a good availability and cost-effectiveness. Therefore, such methods are more practical and promising than chemical or physical detoxification methods (181).

### 4.1 Mycotoxin detoxification by biotransformation or binding

The “detoxification by biotransformation” is a promising new strategy, which is based on microbial degradation of mycotoxins into less toxic compounds. Such a mycotoxin degradation could be also realysed by target microbial enzymes or enzyme preparations. In this regard, the interactions between gut microbiota and mycotoxins can explain the protective effect of the microbiota against toxicity of mycotoxins in some animals, which is often due to degradation of mycotoxins into less toxic compounds or a decrease of their intestinal/ruminal absorption (182, 183). Therefore, development of some probiotics derived from the digestive microflora of some animals are recently initiated for ensuring of mycotoxin degradation (182, 184, 185).

Generally, the mycotoxin degradation by enzymes is not found to be very effective for AFs, ZEA, DON or FUMs and, therefore, the same mycotoxins can be found in beer produced from wheat and maize (186). However, OTA is found to be relatively stable mycotoxin under acid or alkaline conditions, but partial degradation can occur in the presence of some enzymes. Powder of oyster mushroom *Pleurotus ostreatus* was recently studied for possible detoxification of OTA and ZEA by simulation of *in vitro* gastrointestinal digestion in the absence and presence of cornmeal and ground feed. It was found that *Pleurotus ostreatus* has a great potential to detoxify OTA (187) (Table 2).

**Table 2:** Natural mycotoxin degradation/detoxification by biotransformation or binding mycotoxins using target enzymes, yeasts, microorganisms or fungi.

Natural mycotoxin degradation by enzymes, yeasts, microorganisms or fungi	Degradation/detoxification or binding mycotoxins	References
Oyster mushroom *Pleurotus ostreatus*	Detoxification of OTA	Nobre et al. (187)
*Lactobacillus* strains, e.g., *Lactobacillus rhamnosus* strain	Binding AFs	Bovo et al. (62); Afshar et al. (188)
*Saccharomyces cerevisiae* yeast	Binding AFs	Chlebicz and Śliżewska (189)
*Mucor* sp., *Phoma* sp., *Rhizopus* sp. 663, *Rhizopus* sp. 668, *Rhizopus* sp. 710, *Trichoderma harzianum*, *Trichoderma* sp. 639, *Alternaria* sp., *Bacillus subtilis* and target *Sporotrichum strains*	Degradation capacity against AFs is nearly 65–99%	Shantha (190); Kabak and Var (191); Gerbaldo et al. (192); Xia et al. (193)
*Flavobacterium aurantiacum*	Remove AFs	Bhatnagar et al. (194)
*Eubacterium* strain BBSH 797	Degradation of DON to non-toxic de-epoxy-DON	Binder et al. (195)
Yeast strain of *Trichosporon mycotoxinivorans*	Detoxification of OTA and ZEA	Molnar et al. (196)
Yeast strain of *T. mycotoxinivorans*	Degradation of ZEA to non-toxic metabolite ZOM-1	Vekiru et al. (197)
*T. mycotoxinivorans* and *Eubacterium* BBSH 797	*In vivo* degradation of DON, ZEA and OTA	Binder et al. (195); Politis et al. (198); Varga et al. (199)
*Komagataella pastoris*	Detoxification of FUMs	Hartinger and Moll (200)
*Alicyclobacillus* spp	Degradation of PAT in juce	Yuan et al. (201)
Yeast *Saccharomyces cerevisiae*	PAT degradation	Moss and Long (202)
*Lactic acid bacteria* (LAB)	PAT removal	Hatab et al. (203)
*Lactobacillus plantarum*	PAT degradation to hydroascladiol	Hawar et al. (204)
*Byssochlamys nivea* (FF1-2)	PAT degradation	Zhang et al. (205)
Yeasts *Sporobolomyces* sp. strain IAM 13481 and *Rhodosporidium kratochvilovae* strain LS11	PAT degradation to less toxic compounds such as desoxypatulinic acid	Castoria et al. (206); Ianiri et al. (207)
yeast *Rhodosporidium paludigenum*	PAT degradation to desoxypatulinic acid	Zhu et al. (208)
Yeast *Saccharomyces cerevisiae*	PAT degradation to E-ascladiol and Z-ascladiol	Moss and Long (202)
*Gluconobacter oxydans*	PAT degradation to E-ascladiol and Z-ascladiol in apple juice	Ricelli et al. (87)
*Bacillus licheniformis Sl-1, CM 21*	Degradation capacity against OTA is between 35 and 98%	Petchkongkaew et al. (209); Shi et al. (210)
*Acinetobacter calcoaceticus* strain	Degradation of OTA to non-toxic metabolite OTα	Hwang and Draughon (211); De Bellis et al. (212)
*Pediococcus parvulus UTAD 473*	Degradation of OTA (80–90%) to non-toxic metabolite OTα	Abrunhosa et al. (213)
*Lactobacillus plantarum, L. sanfrancisco, L. brevis,* yeast strain *Saccharomyces cerevisiae*	Degradation capacity against OTA is 50–54%	Piotrowska and Zakowska (214); Piotrowska (215)
*Bacillus amyloliquefaciens ASAG1*	Degradation of OTA (98%) to non-toxic metabolite OTα	Chang et al. (216)
*Brevibacterium casei; B. linens; B. iodinum; B. epidermidis*	Degradation of OTA (100%) to non-toxic metabolite OTα	Rodriguez et al. (217)
*Lactobacillus acidophilus*	Degradation of PAT and OTA	Fuchs et al. (218)
*Bacillus licheniformis*	Degradation capacity against AFB1 is about 74%	Petchkongkaew et al. (209)
*B. subtilis*	Degradation capacity against AFB1 is about 85%	Petchkongkaew et al. (209)
*Eubacterium biforme MM11* isolated from swine intestinal microbiota	Degradation capacity against AFB1 and OTA is about 77–100%	Upadhaya et al. (219)
*Eubacterium callanderi, Sphingomonas paucimobilis, S. asaccharolytica, Stenotrophomonas nitritreducens*	Degradation of OTA (95–100%) to non-toxic metabolite OTα	Schatzmayr et al. (51, 220)
*Cupriavidus basilensis ŐR16* strain isolated from soil	Degradation of OTA (100%) to non-toxic metabolite OTα	Ferenczi et al. (221)
*Bacillus subtilis CW 14*	Degradation capacity against OTA is up to 97%	Shi et al. (222)
*Brevundimonas vermicularis B-1, Yeast Yarrowia lipolytica Y-2*	Degradation capacity against OTA is about 84–87%	Wang et al. (223)
*Bifidobacterium bifidum, B. breve, Lactobacillus casei, L. delbrueckii bulgaricus, L. johnsonii, L. paracasei, L. rhamnosus, L. salivarius, L. plantarum*	Degradation of OTA (30–97%) to non-toxic metabolite OTα	Luz et al. (224)
*Aspergillus niger GX312, A. japonicus AX35, A. carbonarius SA332*	Degradation of OTA (83–99%) to non-toxic metabolite OTα	Bejaoui et al. (225)
*Aspergillus tubingensis M036, M074*	Degradation of OTA (up to 95%) to non-toxic metabolite OTα	Cho et al. (226)
*A. niger, A. carbonarius, A. fumigatus, A. clavatus, A. ochraceus, A. versicolor, A. wentii, A. japonicus, Cladosporium* sp.*, P. aurantiogriseum, P. spinulosum, Botrytis cinerea,* isolated from grapes	Degradation of OTA (up to 80%) to non-toxic metabolite OTα	Abrunhosa et al. (227); Bejaoui et al. (228); Valero et al. (229)
*Pleurotus ostreatus*	Degradation of OTA (up to 77%) to non-toxic metabolite OTα	Engelhardt (230)
*Rhizopus stolonifer, R. microsporus, R. homothallicus, R. oryzae, R. stolonifer*	Degradation of OTA (up to 96,5%) to non-toxic metabolite OTα	Varga et al. (231)
*Aspergillus niger M00120*	Degradation of OTA (up to 99%) to non-toxic metabolite OTα	Xiong et al. (232)
*Aureobasidium pullulans AU14-3-1, AU18-3B, AU34-2, LS30*	Degradation of OTA (75–90%) to non-toxic metabolite OTα	De Felice et al. (233)
Yeast strains *Saccharomyces cerevisiae, Kloeckera apiculata, Schizosaccharomyces pombe, Candida pulcherima, Candida friedrichii, Candida intermedia, Lachancea thermotolerans, Cyberlindnera jadinii, Torulaspora delbrueckii*	Degradation of OTA (25–84%) to non-toxic metabolite OTα	Cecchini et al. (234); Angioni et al. (235); Fiori et al. (236); Farbo et al. (237)
Yeast strains *Trichosporon* sp. *DSM 14153, DSM 14156, DSM 14162, 178; Trichosporon mycotoxinivorans MTV, 115; Rhodotorula* sp. *DSM 14155,* 124; *Cryptococcus 118*	Degradation of OTA (80–100%) to non-toxic metabolite OTα	Schatzmayr et al. (51, 220, 238); Molnar et al. (196)
Yeast strain *Yarrowia lipolytica*	Degradation capacity against OTA is about 88%	Yang et al. (239)
Yeast strain *Phaffia rhodozyma CBS 5905*	Degradation of OTA (90%) to non-toxic metabolite OTα and adsorb 23% of OTA	Péteri et al. (240)
Yeast strains *Metschnikowia pulcherrima MACH1, M320; Kloeckera lindneri GAL5; Pichia guilliermondii M8, M29; Rhodococcus erythropolis AR14*	Degradation capacity against OTA is between 26 and 84%	Patharajan et al. (241)
*Stenotrophomonas* sp. CW117, *Luteimonas* sp. CW574, *Silanimonas* sp. CW282, *Lysobacter* sp. CW239 and *Pseudomonas aeruginosa* N17-1	OTA degradation	Chen et al. (181)
*Candida guilliermondii*	PAT degradation	Chen et al. (242)
*Candida famata, Candida guilliermondii, Candida lusitaniae, Cryptococcus laurentii, Kloeckera* spp.*, Rhodotorula glutinis* from Turkish wine-grapes	OTA degradation	Var et al. (243)
*Acetobacter syzygii, Lactobacillus kefiri*	Degradation of AFB1, OTA and ZEA	Taheur et al. (244)
*Actinobacterial strains,* e.g.*, Streptomyces AT10, AT8, SN7, G10, PT1*	OTA degradation (arround 22–52%) and/or adsorbtion (around 16–33%)	Khoury et al. (245)
*Oenococcus oeni*, *Lactobacillus plantarum, Lactobacillus brevis, Leuconostoc mesenteroides, Pediococcus acidilactici* from grape must or wine	OTA degradation	Del Prete et al. (246)
*Oenococcus oeni* isolated from wine	OTA degradation	Mateo et al. (247)
Carboxypeptidase produced by *Bacillus amyloliquefaciens, Phaffia rhodozyma, Acinetobacter* sp. *neg1*	Degradation of OTA to non-toxic metabolite OTα	Péteri et al. (240); Chang et al. (216); Liuzzi et al. (248)
Carboxypeptidase A produced in bovine pancreas	Degradation of OTA to non-toxic metabolite OTα	Pitout (249); Deberghes et al. (250); Abrunhosa et al. (251)
Carboxypeptidase Y produced by *Saccharomyces cerevisiae*	Degradation of OTA to non-toxic metabolite OTα	Abrunhosa et al. (252)
A crude enzyme Ancex	OTA degradation	Abrunhosa et al. (251)
Lipase A produced by *Aspergillus niger*	Degradation of OTA to non-toxic metabolite OTα	Stander et al. (253)
Hydrolase produced by *Aspergillus niger*	Degradation of OTA to non-toxic metabolite OTα	Abrunhosa et al. (254)
Protease A produced by *Aspergillus niger*	Degradation of OTA to non-toxic metabolite OTα	Abrunhosa et al. (251)
A crude metalloenzyme produced by *Aspergillus niger*	OTA hydrolization	Abrunhosa and Venancio (255)
Enzymes polyphenol oxidase or peroxidase	Decrease PAT content in fruits	Chen et al. (256)
Glucose oxidase or peroxidase	Decrease *Alternaria* mycotoxin alternariol (AOH) in fruits	Tittlemier et al. (257); Sun et al. (258)
CotA laccase from *Bacillus licheniformis* ZOM-1	Degradation of ZEA, AFs and AOH	Sun et al. (258)

A lot of microorganisms such as bacteria, actinobacteria, filamentous fungi or yeast were found to effective in OTA degrading or adsorbing. For example, the *Bacillus amyloliquefaciens* ASAG1 (216); *Sphingomonas paucimobilis* 033-1, *S. asaccharolytica* 034-1, *Stenotrophomonas nitritreducens* 041-9 (220); *Stenotrophomonas* sp. CW117, *Luteimonas* sp. CW574, *Silanimonas* sp. CW282, *Lysobacter* sp. CW239, and *Pseudomonas aeruginosa* N17-1 (181) were found to degrade successfully OTA. Some actinobacteria can also adsorb OTA or inhibit its biosynthesis in addition to the possibility to degrade this mycotoxin. For example, actinobacterial strains (*Streptomyces* AT10, AT8, SN7, G10, and PT1) can degrade (arround 22–52%) and also adsorb (around 16–33%) of OTA (245). Some other actinobacterial strains (*Streptomyces* MS1, ML5, and G10) can also inhibit the expression of some biosynthesis genes of OTA in *Aspergillus carbonarius* (245). *Phaffia rhodozyma* CBS 5905 was also able to degrade around 90% of OTA (7.5 μg/mL) within 15 d and adsorbed 23% of OTA (3 μg/mL) within 2 h (240). The most important influence factors of mycotoxin adsorption capacity of microorganisms were found to be cell wall components such as glucogalactans and β-glucans (244) or mannoproteins (259) or β-glucans and mannans (260). However, the mycotoxin adsorption/binding ability by the same microorganism in different culture conditions usually has different manifestations (215, 244, 261). The mycotoxin adsorption by the same microorganism but in different statuses (viable/dead) could be also different (228, 236, 240, 247, 262).

The filamentous fungi such as *Aspergillus niger* GX312, *A. japonicus* AX35, *Aspergillus carbonarius* SA332 (225), in addition to *A. fumigatus*, *A. clavatus*, *A. ochraceus*, *A. versicolor*, *A. wentii*, *Cladosporium* sp., *P. aurantiogriseum*, *P. spinulosum* and *Botrytis cinerea* (isolated from grapes) are also reported to degrade OTA (227–229). In addition, some yeast strains such as *Yarrowia lipolytica* and *Yarrowia lipolytica Y-2* are also reported to be effective in OTA degrading (223, 239). The mechanism of OTA degradation is mainly by its detoxification to the non-toxic metabolite OTα through the hydrolysis of an amide bond via hydrolytic enzymes, such as carboxypeptidase A, carboxypeptidase PJ_1540, protease A, lipase A, ochratoxinase, etc. (181, 252). Another possible mechanism involves OTA degradation via the hydrolysis of the lactone ring (263). Unfortunately, the degradation product in such a case was found to have a similar toxicity to OTA when ingested by rats (264, 265).

Anaerobic *Eubacterium biforme* MM11, isolated from swine intestinal microbiota, was reported to degrade nearly 77–100% of OTA and/or AFB1 in liquid medium or solid corn substrate within 24 h, which suggests that such anaerobic microorganisms could be suitable for development of feed additives (219).

It was recently found that the process of detoxification due to *Lactobacillus* strains, e.g., *Lactobacillus rhamnosus* strain, is mainly due to binding of AFB1 and AFM1 (62, 188). In this regard, “*in vitro”* detoxification of AFB1 by probiotic *Saccharomyces cerevisiae* yeast was seen to be similar to that in *Lactobacillus* strains (189). *Saccharomyces cerevisiae* is such a bacteria, which is most successful at binding to AFB1. Some other strains, e.g., *Mucor* sp., *Phoma* sp., *Rhizopus* sp. 663, *Rhizopus* sp. 668, *Rhizopus* sp. 710, *Trichoderma harzianum*, *Trichoderma* sp. 639, *Alternaria* sp. and target *Sporotrichum strains* are capable to degrade nearly 65–99% of AFB1 (190–193). *Flavobacterium aurantiacum* strain was reported to remove AFB1 (194).

Some other studies reported that *Eubacterium* strain BBSH 797 has capability to transform DON to de-epoxy-DON, which is not a toxic metabolite (195). In a similar way, a yeast strain of *Trichosporon mycotoxinivorans* was reported to detoxify both OTA and ZEA (196). The same *T. mycotoxinivorans* strain was found to cleave OTA into phenylalanine and OTα, which does not have toxic effect on animals (220). ZEA was found to be metabolized by the same strain into the nontoxic metabolite ZOM-1, which is without estrogenic properties (197). In following experimental studies, the both strains *T. mycotoxinivorans* and *Eubacterium* BBSH 797 were reported to destroy the same mycotoxins “*in vivo”* (198, 266). It is worth to mention, that *Trichosporon mycotoxinivorans* strain was not only reported to degrade OTA, but also it can meet the prerequisites for usage as an animal feed additive based on a European Food Safety study (199). Due to the excellent OTA-detoxification performance of *T. Mycotoxinivorans* (MTV, 115), it was made into a commercial product named Mycofix® Plus^MTVINSIDE^ by Biomin GmbH (Austria) (267). Experimental studies with chicks confirmed that OTA induced toxic effects, e.g., decreased body weight gain, poor feed conversion ratio, increased levels of serum lactate dehydrogenase, aspartate aminotransferase, and γ-glutamyltranspeptidase, in addition to pathological changes in bursa of Fabricius, spleen, liver, and kidney, were significantly attenuated by the same commercial product (267). A similar example of such a degradation presents the gene coding for a carboxylesterase, which has been isolated from a soil microorganism and subsequently cloned into *Pichia pastoris*, later renamed to *Komagataella pastoris.* The same enzyme is possible to detoxify FUMs in the gastrointestinal system of pigs into some non-toxic compounds (200).

Some microorganisms having a good OTA adsorption ability also have great application prospects in food or feed industries (237, 268, 269). For example, within a 90-day fermentation process, OTA (4 μg/mL) was found to be adsorbed of *S. cerevisiae* by 73, 85, and 90% in white, rose, and red wine musts, respectively (268). Similarly, immobilized *Candida intermedia* 253 yeast cells into magnetic calcium alginate beads were reported to be effective in adsorbing OTA in commercial grape juice, and more than 80% of OTA (0.02 μg/g) was adsorbed within 48 h of incubation (237).

Another good example is the licensed probiotic preparation, including *L. paracasei* LOCK 0920, *L. plantarum* LOCK 0945, *L. brevis* LOCK 0944, *S. cerevisiae* LOCK 0140, and *Yucca schidigera* extracts, which was used to decrease OTA content in broiler feed (270), as the contamination levels of 1 ppm and 5 ppm OTA were reduced by 73 and 55% in feed within 6 h fermentation with the probiotic preparation, respectively. A yeast strain *Kluyveromyces marxianus* C2, isolated from pig feces, was also found to reduce 82.3% of OTA (0.5 μg/mL) in YPD medium and 83.7% of OTA (0.082 μg/g) in moldy corn feed, respectively (181).

Various biological methods (using target biological agents) were developed for decreasing of PAT or *Alternaria* mycotoxin contamination in fruits and derived products, e.g., yeast fermentation and adsorption, degradation by enzymes, degradation by lactic acid *bacteria* (LAB), etc. (201–204, 271). Such biodegradations do not impart significant changes in quality of the product, but some further studies are necessary for clarifying the involved mechanisms and the possibility of safe usage of such methods in order to elaborate the required parameters for their successful usage in the fruit and/or juice industry.

A study reported a successful removal of PAT in some fruit or fruit products is described by Yuan and collaborators, who have found that the contamination level of PAT in juice can decrease nearly 88% by usage of 49 g/L inactivated *Alicyclobacillus* spp. (201). In another study, it was reported that PAT was successfully removed by usage of strain *Byssochlamys nivea* (FF1-2), which cannot produce PAT (205). Some other *in vitro* experiments showed that the yeasts *Sporobolomyces* sp. such as strain IAM 13481 or *Rhodosporidium kratochvilovae* strain LS11 have possibility to resist PAT and to ensure its degradation to less toxic metabolites such as ascladiol and desoxypatulinic acid (207).

The mechanisms of biological removal of PAT are considered to be as follow: the biosorption of PAT by microbial cells (272, 273), degradation by some enzymes produced by target microorganisms (208), destroying the functional properties of PAT (274, 275). Also, another explanation would be that the availability of PAT in growth media can provoke the production of PAT-degrading enzymes by PAT-resistant yeasts (208, 276). In this regard, it was found that PAT can be degraded by *Saccharomyces cerevisiae* and *Gluconobacter oxydans* to E-ascladiol and Z-ascladiol (87, 202), by *Candida guilliermondii* (242); by *L. plantarum* - to hydroascladiol (204), by *Rhodosporidium paludigenum* - to desoxypatulinic acid, which are not or less toxic (208).

According to some authors, enzymes play an important role in detoxification or degradation of PAT in juices prepared by pome fruit (207) and some antioxidative enzymes are main factors in the process of elimination of reactive oxygen species (206).

Some enzymes, e.g., polyphenol oxidase or peroxidase were found to decrease PAT content in fruits (256). In this regard, polyphenol oxidase produced by extraction from apples was found to decrease significantly PAT content in apple juice. Enzymes such as glucose oxidase or peroxidase were also reported to decrease *Alternaria* mycotoxin in fruits. In this regard, peroxidase which is produced by extraction from horseradish was found to decrease the content of *Alternaria* mycotoxin alternariol (AOH) in tomatoes (257). Glucose oxidase, which is synthesized by *Aspergillus niger* was found to decrease AOH content in apples, whereas CotA laccase from *Bacillus licheniformis* ZOM-1 was found to degrade ZEA, AFs and AOH (258).

Enzymes are also used for OTA degradation. Proteolytic enzymes such as trypsin, α-chymotrypsin and carboxypeptidase A were reported to hydrolyse OTA for the first time in 1969, but among them carboxypeptidase A was found to be more effective (249). A crude enzyme Ancex is found to be more effective in OTA degradation as compared to some commercially purified enzymes like Protease A, Pancreatin or Prolyve PAC (251). Another crude metalloenzyme isolated from *Aspergillus niger* was found to have a much higher OTA hydrolytic activity as compared to the carboxypeptidase (255). Similarly, the crude enzymes of *Aspergillus tubingensis* (M036 and M074) were found to remove about 90–97% of OTA at defined pH 5 and 25°C (226). The purified recombinant ochratoxinase was also found to hydrolyze OTA more efficiently than carboxypeptidase A at an optimal pH and temperature (277).

It seems that a large number of microorganisms, which have a good OTA degradation and/or adsorption ability, in addition to some OTA degradation enzymes isolated or cloned from such microorganisms or animal pancreas, received also great application prospects in food or feed industry.

Nowadays, biodegradation was found to be an emerging and promising new strategy for control of mycotoxins, because of the outstanding efficiency without possible pollution of the treated products. It was found that a lot of fungi or bacteria can degrade mycotoxins in feed (191–193). However, some futurer research efforts are required to clarify the mechanisms, which are involved in such a degradation or detoxification and to define and isolate enzymes responsible.

### 4.2 Natural antagonists against target fungi as a valuable alternative to conventional fungicides for preventing mycotoxin contamination

Another practical way to prevent feeds/foods from mycotoxin contamination is to use some target microbial antagonists, which could be a valuable alternative to conventional fungicides. In this regard, a special attention should be paid to the development of target bioeffective technologies for preventing the growth of toxigenic fungi, which are based on the natural bioeffective microbial aggents. In this regard, *Bacillus subtilis* strains, were found to suppress the fungal growth of *Fusarium* strains (48). Also, it was reported that non toxigenic *Aspergillus flavus* strains can decrease AFs content in the treated feedstuffs or food commodities (278), because such strains grow on the same ecological niche as mycotoxigenic strains and can displace them. Therefore, the spores of such non toxigenic *A. flavus* strains inoculated on various grains such as barley or sorghum can be used for prevention excessive AFs contamination in feeds/foods. The introduction of atoxigenic *A. flavus* strains, which are capable to replace fully AFs producing strains, would be a useful strategy for decreasing preharvest AFs contamination levels of crops (279) (Table 3).

**Table 3:** Natural antagonists such as yeasts, microorganisms, fungi or biologically active substances against the growth of target mycotoxin producing fungi.

Natural antagonists such as yeasts, microorganisms, fungi or biologically active substances against target mycotoxin producing fungi	Suppression the growth of following fungi and subsequent mycotoxin production	References
*Lactobacillus plantarum*	Suppression the fungal growth of *Aspergillus parasiticus* and *Penicillium expansum* and subsequent production of AFs and PAT	Luz et al. (275)
*Bacillus subtilis* strains	Suppression the fungal growth of *Fusarium* strains and subsequent FUMs production	Jouany (48)
*Actinobacterial strains,* e.g.*, Streptomyces* MS1, ML5 and G10	Inhibition of the expression of some biosynthesis genes of OTA in *Aspergillus carbonarius*	Khoury et al. (245)
Non toxigenic *Aspergillus flavus* strains	Displace mycotoxigenic strains by biocompetition and decrease AFs content in the feedstuffs or food commodities	Cole and Cotty (278); Moral et al. (279)
*Rhodotorula glutinis* LS11	Suppression the fungal growth of *Penicillium expansum* and subsequent production of PAT	Castoria et al. (280)
*Pichia ohmeri*	Suppression the fungal growth of *Penicillium expansum* and subsequent production of PAT	Coelho et al. (281)
*Pichia caribbica* yeast	Suppression of blue mold rot and subsequent PAT production in apples	Cao et al. (282)
*Candida sake CPA-2 and Pantoea agglomerans CPA-1*	Suppression the fungal growth of *Penicillium expansum* and subsequent production of PAT	Morales et al. (283)
Yeast species of ascomycota (*Pichia ohmeri 158 and Candida guilliermondii P3*)	Suppression the fungal growth of *Penicillium expansum* and subsequent production of PAT	Coelho et al. (284)
*Candida membranifaciens* and *Torulaspora delbrueckii*	Suppression the fungal growth of *Penicillium expansum* and subsequent production of PAT	Farahani et al. (285); Ebrahimi et al. (286)
Dip treatment of apples with suspension of microorganisms *Pseudomonas fluorescens* or *Bacillus subtilis*	Suppression the fungal growth of *Penicillium expansum* during the cold storage and subsequent production of PAT on apples	Narayanasamy and Narayanasamy (287); Wallace et al. (288)
*Pseudomonas syringae*	Suppression of postharvest fungal growth of *Botrytis cinerea* and *P. expansum* on apples and subsequent production of PAT	Zhou et al. (271)
Predacious yeast *Saccharomycopsis schoenii*	Biological control of fungal growth of *Penicillium italicum, P. digitatum* and *P. expansum* by true predation	Pimenta et al. (289)
Polyphenols, flavonoids, carotenoids and silymarin	Suppression the fungal growth of *Aspergillus flavus* and subsequent production of AFs	Zhou et al. (64); Shen et al. (63); Valencia-Quintana et al. (83)
The essential oils such as cinnamon and clove oil	Decrease PAT contamination in apples	Sivakumar and Bautista-Baños (84)
Plants extracts of essential oils and monoterpenoids	Suppression the fungal growth of *Aspergillus terreus, Fusarium oxysporum, Penicillium expansum* and *Verticillium dahliae* and subsequent mycotoxin production	Kadoglidou et al. (85)
Garlic extract and garlic vapor exposure of apples	Suppression the fungal growth of *Penicillium expansum* and subsequent production of PAT	Ikeura et al. (86)
*Lentinula edodes* lyophilised filtrates	Stimulation of *A. parasiticus* anti-oxidant enzymes production (superoxide dismutase, catalase, glutathione peroxidase) and suppression of AFs production by *A. parasiticus*	Reverberi et al. (88)
Vanillic acid	Inhibition of OTA production and growth of *Aspergillus* species.	Palumbo et al. (89)

The addition of bioeffective microorganisms that can suppress *P. expansum* growth or destroy PAT metabolism is found to be useful biocontrol technique for preventing PAT contamination in the prolonged storage of some sensitive products prepared by fruits. The accumulation of PAT in fruit at storage time could be also prevented by the effective control for possible decay of fruit, which is usually provoked by *P. expansum*. In this regard, cells of *Rhodotorula glutinis* LS11 were reported to decrease contamination levels of PAT and destroy it in “*in vitro”* study (280). It is reported, that PAT content can be decreased by nearly 83% after 2 consequent days following the incubation with *Pichia ohmeri* and after 15 days PAT was found to be undetectable (281), whereas inoculation of *Pichia caribbica* yeast can significantly decrease PAT content in apples after 15-day incubation period (282). A significant decrease in accumulation of PAT was also demonstrated by using some other fungal suppressing agents such as *Candida sake CPA-2* and *Pantoea agglomerans CPA-1* (283) or some yeast species such as *Pichia ohmeri 158 and Candida guilliermondii P3* (284). Such a decrease of PAT content was assumed to be a consequence of the protection of fruit against contamination of toxigenic *P. expansum* species or by absorption, but not metabolization or degradation of PAT (290).

Some antagonistic microorganisms/yeasts or extracts of such microorganisms/yeasts inhibiting the growth of fungi and subsequent PAT production are reported for apples and laboratory cultures, e.g., *Pichia caribbica* (282) or *Candida membranifaciens* and *Torulaspora delbrueckii* (285, 286). Such a suppression of mycotoxin production by *P. expansum* via using antagonist yeast inoculation is supposed to be due to the the suppression of the growth of *P. expansum* (291). Some cultures of LAB or LAB-supernatants, which are free of cells were reported to suppress PAT-production by *P. expansum* or other fungi (275).

Some microorganisms, e.g., *Pseudomonas fluorescens* or *Bacillus subtilis* were reported to be useful in decreasing PAT contents in apples (287). For example, the dip treatment of McIntosh and Spartan apples with cell suspension of *Pseudomonas fluorescens* realized before the dip treatment with spore suspension of *P. expansum* was found to suppress the fungal growth at the time of commercial cold storage of apples. The same protection was found to be similar to that of commercially availble fungicides (288). Similarly, a powerful control of the fungal growth of *Botrytis cinerea* and *P. expansum* was demonstrated by usage of some isolates of *Pseudomonas syringae* during 28 days of storage (271).

Such inhibition by some bioactive agents (yeast, fungi or bacteria, suppressing the growth and PAT production by fungi) could be also explained by the possible competition for the necessary nutritients or the space available as well as by some generated bioactive (antagonistic) substances of antagonistic agents, which can suppress the germination of spores and development of mycelium of the fungi or by true predation (288, 289).

## 5 Other miscelaneous natural antidotes or vitamins given as supplements in the diet to combat mycotoxicoses

Another possibility for reducing toxicity of mycotoxins is to know in depth the particular mechanisms of their toxicity in order to find specific antidotes or vitamins, which could be used as supplements to the diet for preventing the specific toxicity of each separate mycotoxin (2, 91). For example, some of the toxic properties of OTA are attributed to the structural similarity of this mycotoxin with phenylalanine, which can explain the subsequent suppression of protein synthesis as a result of competition for target t-RNA (292). Unfortunately, the possible protection of phenylalanine is studied mostly by *in vitro* experimental investigations using yeast or bacteria, but only a few experimental studies were performed with animals/poultry or laboratory animals (8, 16, 97, 99, 100).

Such a protection of phenylalanine was seen against OTA-provoked biochemical and pathological changes in rats. In the same experimental study, the number of OTA-provoked tumors in phenylalanine-supplemented rats treated additionally by 10 ppm OTA was comparable to that in the group treated with two times lower OTA concentrations, which suggests for such a protection of phenylalanine against OTA-provoked tumors (99, 100). Such a protection of phenylalanine was also found against OTA-provoked malformation in rats given phenylalanine as supplement to the diet (16).

The protective effect of phenylalanine against OTA-induced immunosuppression in humoral immunity was reported to be a consequence of the improved protein synthesis, which is usually impaired by OTA and to a subsequent improvement of OTA-provoked delay of the division of immune cells (97). Such a mild protection of phenylalanine against OTA exposure was also seen in the eggs production of laying hens (8).

It was also found, that 300 ppm ascorbic acid supplementation to the diet of laying hen contaminated with 3 ppm OTA can decrease the toxic effect of OTA on the production and weight of the eggs (151, 152) (Table 1).

Some vitamins such as ascorbic acid and/or vitamin B, are also reported to be useful for PAT degradation (32). Ascorbic acid and ascorbate which are present naturally in apples, were reported to be able to decrease PAT-content in apple juice (30, 153, 154). This degradation of PAT by ascorbic acid was found to be very strong and rapid in the presence of light and oxygen (30).

Another similar protection is reported about the combination of citric acid and sodium bicarbonate given as supplements to the apple juice, which are found to decrease PAT content (155).

A dose-dependent protection of Vitamin E against immunotoxic effect of OTA was also reported in chicks, but such a protection was not significant when the chicks were exposed to OTA in contamination levels above 2 ppm (103).

## 6 Some changes in the diet of animals as a means to combat some mycotoxicoses

Some changes in the diet such as low carbohydrate intake, increased protein content, calorie restriction or restriction of the dietary fats were also reported to be useful as a means to combat some mycotoxicoses. In this regard, some recent studies reported that low carbohydrate exposure and the restriction of calories could be useful in amelioration of toxic effects of AFs, and the increased protein content in the diet could also help for detoxification of AFs (32, 293).

However, there are some contradictions in the results concerning the importance of dietary fats on AFs content, but it is suggested that the increased fat in the diet could partially promote the retention of AFs in the body as compared to a diet containing low fat (293).

Another possibility to safely utilize feedstuffs contaminated with mycotoxins and to prevent toxic actions of mycotoxins on farm animals or poultry is to give such feedstuffs to some animal species or poultry that are less sensitive to a target mycotoxin. For example, ruminants can utilize safely OTA-contaminated feedstuffs, because the same animals are not so susceptible to OTA and can hydrolyze it via the rumen to the nontoxic metabolite ochratoxin α (OTα) (294).

It is important to emphasize, that any piece of knowledge about the metabolism of mycotoxins and the way of detoxification or elimination of each mycotoxin in each kind of animal is of particular importance for finding an appropriate way to reduce the toxicity of each particular mycotoxin.

## 7 Concluding remarks

Having in mind, that traditional chemical methods for mycotoxin decontamination have some significant disadvantages such as low efficiency, loss of taste, decreased nutritive value of the feedstuffs/foods, some harmful side effects on the health and the high cost of the required equipment, their wide application (excluding ammoniation) is limited and considered impractical and even potentially harmful for extensive use in the real practice (27). On the other hand, clay binders are rarely effective against most of mycotoxins, with exception of AFs and PAT, giving way to natural organic binders, which are more effective against multi-mycotoxin contamination of forages and also are well biodegradable, preventing subsequent contamination of the environment.

Therefore, herbal/plant additives, enzymatic preparation, natural antioxidants or adsorbents, natural organic binders, biological agents with fungicidal properties, microorganisms and yeasts are recently prefered as more practical and safe ways for mycotoxin decontamination. On the other hand, biological detoxification methods provide a better food safety and preserved flavor of treated food/feed, accompanied with preserved nutritional quality and organoleptic properties of treated food/feed. Therefore, these methods are more practical and promising than chemical or physical detoxification methods, showing also a good availability and cost-effectiveness. In this regard, some additional research efforts are required to reveal their real addvantages towards the traditional methods for mycotoxin decontamination, and to clarify the intimate mechanisms of detoxification and/or enzymes involved in it.

Nowadays, biological supplements containing antagonistic microorganisms, target enzymes, yeasts or plant extracts or even some natural antioxidants are considered to be a good alternative to conventional fungicides, and can promote “detoxification by biological transformation or degradation of mycotoxins,” which is a promising new strategy for control of mycotoxin content due to its high efficacy and environmental safety. The most important advantages of such biological supplements are their easy utilization or easy excretion by animals/humans and the absence of any toxic effects. Biological supplements such as target natural antioxidants, have been reported to be very effective and safe in postharvest fungal control and suppression of production of PAT, AFs and OTA. Therefore, some emerging bioactive supplements receive a great attention from the commercial feed industry, because of providing a safe control of mycotoxin content or decreasing mycotoxin bioavailability. However, finding high-performance strains, which could simultaneously absorb or biodegrade multiple mycotoxins will be an important task for future research.

Herbs or herbal extracts (using polar solvents for extraction) from *T. cordifolia, Gl. glabra, W. somnifera, S. marianum* and/or Silymarin or natural plants such as turmeric powder, could also serve as good protectors, together with some natural mycotoxin-binders, and could decrease deleterious effects of mycotoxins, providing a better utilization of mycotoxin-contaminated fodder and a higher weight gain or eggs production of mycotoxin-exposed commercial poults or animals. The possible economic loss from scrapping of mycotoxin containing fodder or from loss of body weight of animals or poultry could be safely avoided only with minimal costs for purchasing the herbs or herbal products. In this regard, some additional studies and efforts are required for possible introduction of such a protection in large scale use in the real practice, ensuring a safe utilization of mycotoxin-contaminated feedstuffs.

Moreover, natural bioactive substances in plant extracts or volatiles (e.g., some phenolic compounds, polyphenols, flavonoids, silymarin or carotenoids) may also act as antifungal agents and are found to be very effective against fungal growth in fruits at postharvest time and their application for such a purpose is absolutely safe. Such compounds are found to suppress strongly the fungal growth of *A. flavus* and AFs contamination of fodder, and could be also used in the practice. Therefore, any kind of knowledge about the metabolism and the intimate mechanisms of detoxification or excretion of each kind of mycotoxin in each kind of animals or poults is very valuable for providing a safe utilization of mycotoxin containing fodder without increasing the health hazard.

## Author contributions

SS: Investigation, Visualization, Writing – original draft, Writing – review & editing.
